# Shifting the Balance: Mitochondrial Heteroplasmy as a Driver of Cardiac Disease

**DOI:** 10.1161/CIRCRESAHA.126.328769

**Published:** 2026-08-14

**Authors:** Andriana Papadaki, Itamar Braga Dias, Christoph Maack, Peter van der Meer, Nils Bomer

**Affiliations:** 1 Department of Cardiology, University Medical Center Groningen, University of Groningen, the Netherlands (A.P., I.B.D., P.v.d.M., N.B.).; 2 Department of Translational Science, University Clinic Würzburg, Germany (C.M.).; 3 Department of Internal Medicine I, University Hospital Würzburg, Germany (C.M.).

**Keywords:** biopsy, cardiomyopathies, DNA, mitochondrial, heart failure, heteroplasmy

## Abstract

Mitochondrial heteroplasmy represents a fundamental determinant of mitochondrial function and disease, yet its consequences vary across different tissues. Although mitotic tissues possess mechanisms, such as cell division and mitochondrial turnover, to dilute or remove deleterious variants, postmitotic tissues lack this renewal capacity and are disproportionately vulnerable. Neuromuscular and neurodegenerative disorders have illustrated the impact of heteroplasmic mutations, but the (postmitotic) heart remains underexplored. Current reliance on blood-derived samples provides only an indirect view of cardiac heteroplasmy, highlighting the need for alternative approaches, such as endomyocardial biopsies and human induced pluripotent stem cell–derived cardiomyocytes. Expanding cardiac-focused research is essential for identification, clarifying pathogenesis, improving risk stratification, and guiding patient monitoring. Emerging therapies, including mitochondrial transplantation and mitochondrial-targeted DNA editing, demonstrate potential to modulate heteroplasmy and restore equilibrium. Integrating these strategies with precision medicine will be vital for addressing tissue-specific vulnerabilities. Ultimately, bridging the gap in cardiac heteroplasmy research will be critical for translating basic mitochondrial biology into meaningful clinical advances.

Mitochondria originated from an ancient endosymbiotic event in which an α-proteobacterium was engulfed by an archaeal host cell, ultimately evolving into the first eukaryotic lineage.^1^ Through symbiosis, the prokaryote (endosymbiont) became dependent on the host for protection and nutrients, whereas the host relied on the endosymbiont’s ability to produce energy through cellular respiration, a process that provided a significant evolutionary advantage. Over time, the endosymbiont evolved into the organelle we now call a mitochondrion, progressively losing the majority of its genome and developing a complex protein import system and integrating into the host’s cellular machinery.^2^ Mitochondria are currently essential bioenergetic organelles and the primary generators of cellular ATP in eukaryotes, mainly through oxidative phosphorylation (OXPHOS).

Despite their divergence from the ancestral bacterium, modern mitochondria retain several modern prokaryotic features. For example, they harbor their own prokaryote-like circular genome of ≈16.6 kb.^3^ These organelles are thought to have lost most of the genes once carried by their prokaryotic ancestor,^4^ with the mitochondrial DNA (mtDNA) currently encoding just 37 genes: 13 encode proteins essential for the OXPHOS system, 22 encode transfer RNAs, and 2 encode ribosomal RNAs.^3^ The proper function of mitochondria relies on the coordinated expression of ≈1500 genes encoded in the nDNA.^5^ Although present-day mitochondria do synthesize a few of their own proteins translated from their own mtDNA, which is present in hundreds to thousands of copies per cell, the vast majority of the proteins they require for normal function are now encoded in the nDNA, which exists as a single copy.^6,7^ Within the OXPHOS system, mtDNA encodes protein components of complexes I, III, IV, and V of the electron transport chain embedded in the inner mitochondrial membrane.^8^ The electron transport chain is central to mitochondrial function as it generates the proton gradient that drives ATP synthesis.^9^ The mtDNA-encoded protein components of the electron transport chain, together with nDNA-encoded subunits, comprise assemblies of varying size.^10^ For instance, complex I is the largest component of the electron transport chain, composed of 45 subunits, of which 7 are encoded by the mtDNA, whereas the remaining 38 derive from the nDNA.^11,12^

This dual genetic control of mitochondrial proteins means that pathogenic variants in either the mitochondrial or nuclear genome can give rise to mitochondrial dysfunction. Indeed, many components of the OXPHOS system are encoded by nDNA, and pathogenic mutations in these genes account for a substantial proportion of mitochondrial disease.^13,14^ However, the focus of this review is on pathogenic variants within the mtDNA itself, which uniquely give rise to the phenomenon of mitochondrial heteroplasmy. The multicopy nature of mtDNA allows wild-type and mutant variants to coexist within the same mitochondrion. This coexistence of wild-type and mutant mtDNA is known as mitochondrial heteroplasmy.^15^

The impact of mitochondrial heteroplasmy is amplified by the fundamental differences between the nDNA and mtDNA. The integrity of nDNA is protected by histones, efficient DNA repair mechanisms, and replicated with high fidelity. In contrast, mtDNA is packaged less tightly and lacks many canonical DNA repair mechanisms.^16^ As a result, the relative proportions of wild-type and mutated mtDNA become critical for mitochondrial function. When the proportion of mutant variants surpasses a critical threshold, typically between 60% and 90%, mitochondrial dysfunction ensues,^17^ and this threshold significantly varies between different tissues.^18^ Whether mitochondrial dysfunction occurs due to the excess pathogenic mtDNA variants or the lack of wild-type mtDNA is yet to be understood. Accordingly, this review provides a critical and directional synthesis aimed at identifying knowledge gaps and methodological constraints in the study of mitochondrial heteroplasmy in the heart. The clinical and translational significance of mitochondrial genetic variation in the cardiovascular system has recently been formally recognized in an American Heart Association Scientific Statement, which comprehensively outlines the current understanding of the role of mitochondrial genetics in cardiovascular pathobiology and highlights key knowledge gaps that remain to be addressed.^19^

## Causes of Mitochondrial Heteroplasmy

**Figure 1.**
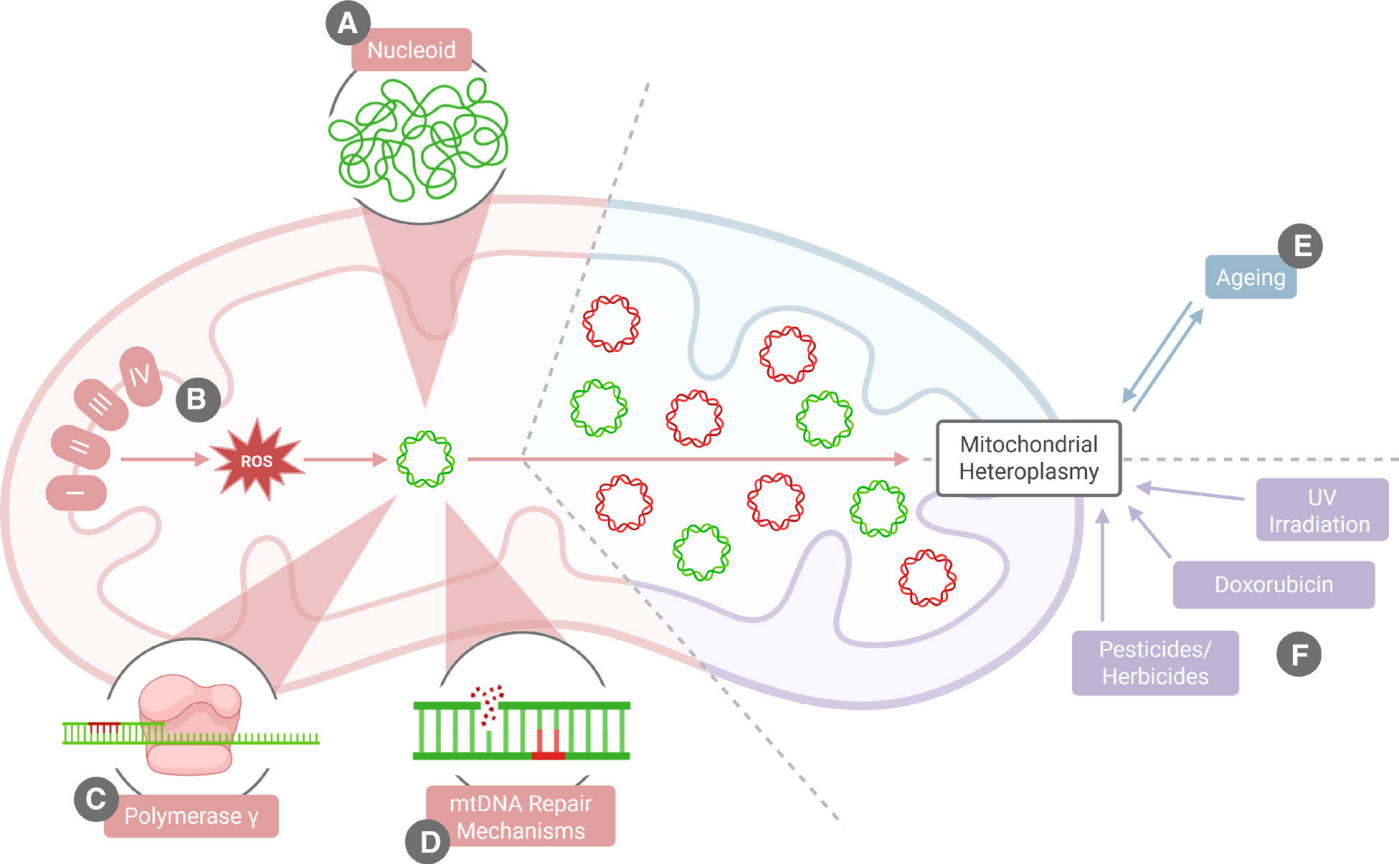
**Schematic overview of the major contributors to mitochondrial heteroplasmy.** Multiple intrinsic and extrinsic factors influence the accumulation of mutant mitochondrial DNA (mtDNA; red mtDNA molecules) within cells (wild-type mtDNA variants are depicted in green). Key intrinsic mechanisms include (**A**) loose mtDNA packaging in nucleoids, (**B**) proximity to reactive oxygen species (ROS) produced from the electron transport chain, (**C**) low fidelity of Pol γ (polymerase gamma) during mtDNA replication, and (**D**) limited canonical DNA repair mechanisms mainly restricted to base excision repair. Extrinsic contributors include (**E**) aging, which both drives and is accelerated by heteroplasmy reflecting a bidirectional relationship, and (**F**) environmental and pharmacological stressors, as well as ultraviolet (UV) irradiation. Collectively, these factors create a dynamic heteroplasmic landscape.

Mitochondrial heteroplasmy arises from a combination of inherited and acquired factors. At baseline, heteroplasmy is maternally transmitted, as mtDNA is almost exclusively inherited through the oocyte.^20,21^ This means that the offspring begins life with a preexisting distribution of wild-type and mutant mtDNA variants, determined by the maternal germline.^22^ However, this inherited mitochondrial heteroplasmy is exacerbated through the accumulation of de novo mutations in mtDNA over an individual’s lifetime (Figure 1).^23^

In both mitotic and postmitotic tissues, heteroplasmy shifts are driven by mutations that emerge because of the mtDNA’s intrinsic fragility. Unlike nDNA, the mtDNA is not organized in nucleosomes by histones, but it is less densely packaged in mitochondrial nucleoids (Figure 1A), bound by the TFAM (mitochondrial transcription factor A) as its primary component.^24,25^ Therefore, the mitochondrial nucleoids render the mtDNA directly more exposed to reactive oxygen species (ROS) generated by the adjacent electron transport chain within the mitochondria (Figure 1B).^26^ Excessive ROS production leads to mtDNA damage and ROS-induced de novo mutations.^27^ Over time, this leads to the accumulation of more pathogenic mtDNA variants within the same cell. Although nDNA benefits from robust repair pathways such as nucleotide excision repair, mismatch repair, base excision repair, and homologous recombination, mitochondria lack most of these mechanisms.^28^ MtDNA primarily relies on base excision repair^29^ and limited evidence suggests the presence of microhomology-mediated end joining^30^ and homologous recombination^31^ within the mitochondria (Figure 1D). The limited repair capabilities of mtDNA mean that the ROS-induced de novo mutations are more likely to persist over time.

A further strong contributor to mutational burden is the fidelity of mtDNA replication of DNA Pol γ (polymerase gamma; Figure 1C).^32^ Although it provides proofreading via its exonuclease activity, its activity is oxidation sensitive.^33^ Under oxidative stress, the proofreading efficiency of Pol γ plummets, and mtDNA fidelity is reduced. This allows for the expansion of point mutations and mtDNA deletions over time.^34^ Notably, this replication process is not confined to mitotic cycles. mtDNA undergoes continuous replication throughout the cell cycle, even within postmitotic tissues.^35^ This relaxed and continuous mode of replication ensures that mutation-inducing events persist long after cell division has ceased, allowing for further accumulation of somatic heteroplasmic variants.

Together, these intrinsic features continuously shift the mitochondrial heteroplasmic balance of the mtDNA further from the wild-type state. Over time, aging (Figure 1E), characterized by a higher oxidative stress environment, can also further exacerbate this mutational burden primarily through replication errors.^36,37^ In postmortem human brain tissues, Klein et al^38^ reported age-related accumulation of heteroplasmic mtDNA mutations. Their analysis from cross-sectional comparison between individuals aged 70 to 100 years specifically revealed an average of 1.6% increase per year.^38^ However, mitochondrial heteroplasmy and aging engage in a reciprocal relationship, with each influencing the other in a reinforcing cycle (Figure 1E). The link between mtDNA damage and aging was demonstrated in a murine model expressing a defective Pol γ.^39^ The authors observed an increased number of somatic mtDNA mutations, which led to an accelerated aging phenotype.

Beyond aging, increasing evidence suggests that external factors, such as environmental toxins, pharmaceuticals, and ultraviolet irradiation (Figure 1F), may also contribute to mtDNA instability.^40^ As previously described, mtDNA is inherently more fragile than nDNA.^16^ Its intrinsic vulnerabilities, including loose packaging, low replication fidelity, and limited DNA repair mechanisms, make the mtDNA particularly susceptible to external factors. For example, both ultraviolet A and ultraviolet B irradiation have been associated with oxidative mtDNA lesions and impaired mitochondrial function, with ultraviolet A primarily promoting ROS-induced de novo mutations and ultraviolet B causing direct mtDNA damage.^41^ In addition, environmental toxicants, such as pesticides and herbicides, can induce excessive mitochondrial ROS production, disrupt ETC (electron transport chain) activity, and promote direct mtDNA damage.^42^ Similarly, some pharmaceuticals, for example, doxorubicin, are well recognized for inducing mitochondrial oxidative stress and mtDNA damage and impairing bioenergetics.^43,44^ Collectively, these exposures may contribute to the accumulation of mtDNA mutation; however, their direct connection with mitochondrial heteroplasmy is still poorly understood and requires further study.

## Consequences of Mitochondrial Heteroplasmy

Mitochondrial heteroplasmy manifests differently across various tissues depending on their inherent metabolic demands.^45^ Certain tissues, like the heart and brain, are among the most energetically demanding in the body.^46^ This high energy demand requires robust mitochondrial function to meet continuous ATP needs. This is achieved not only by relying on mitochondrial OXPHOS but also through maintaining a reserve respiratory capacity that can be recruited during bioenergetic stress.^47^ When energy demand rises, and this reserve is consumed, cells are left more vulnerable to additional defects, such as mitochondrial heteroplasmy. This bioenergetic stress can be ultimately defined as the mismatch between a tissue’s ATP demand and its mitochondrial ATP production capacity^48^ that strongly amplifies the functional consequences of mitochondrial heteroplasmy. Therefore, even at modest mutant loads, mitochondrial heteroplasmy can lead to severe neuromuscular diseases associated with mtDNA mutations.^46^ Here, pathogenic thresholds of mtDNA heteroplasmy vary considerably between specific mtDNA mutations. For example, mitochondrial encephalomyopathy, lactic acidosis, and stroke-like episodes (MELAS) is a mitochondrial disorder primarily caused by the m.3243A>G (transfer RNA^Leu(UUR)^) point mutation (Table).^64^ When the pathogenic variants exceed only 60% of heteroplasmy levels, patients with MELAS manifest stroke-like episodes, muscle weakness, and seizures.^17,65^

**Table.**
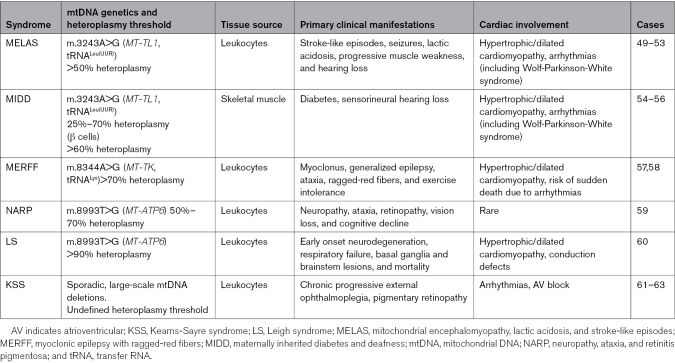
Clinical Spectrum of Mitochondrial Heteroplasmy–Associated Neuromuscular Disorders

Alongside high metabolic needs, a tissue’s proliferative capacity critically dictates how mitochondrial heteroplasmy will manifest and evolve over time. Mitotic tissues, such as the skin, can dilute or purge the mutation burden on the mtDNA through a series of different mechanisms.^45^ In contrast, postmitotic tissues, including the brain, skeletal muscles, and the heart, lack such mechanisms. This, in combination with their large bioenergetic stress, renders them particularly vulnerable to the accumulation of heteroplasmic mtDNA variants.

### Mitotic Tissues

**Figure 2.**
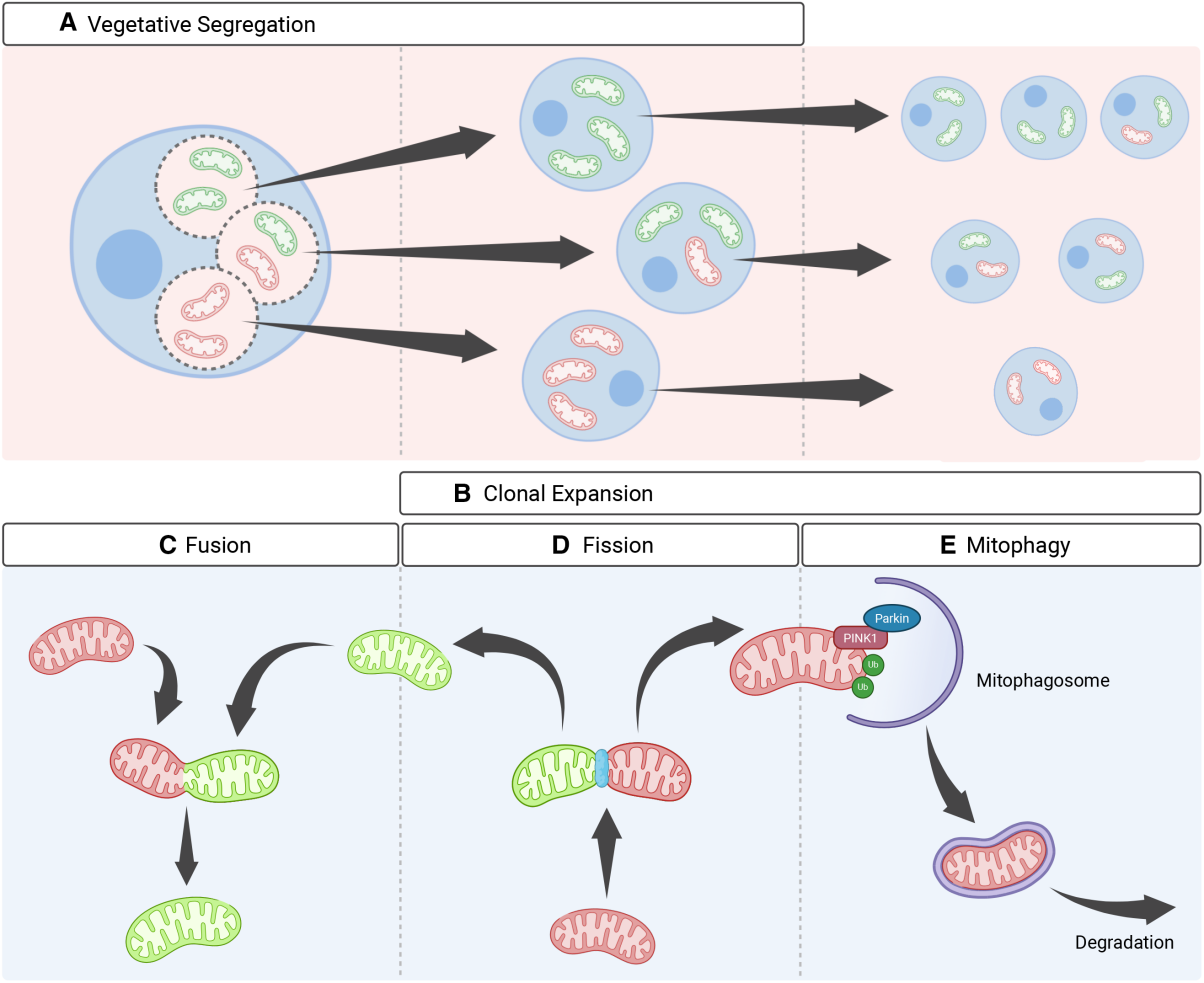
**Schematic overview of the intrinsic mechanisms that protect mitotic tissues from the accumulation of pathogenic heteroplasmic mitochondrial DNA (mtDNA) variants. A**, During cell division, vegetative segregation of mtDNA leads to the random distribution of wild-type (green) and pathogenic (red) mitochondria across daughter cells, reducing the mutant load over successive generations. **B**, Clonal expansion favors the proliferation of lineages with lower levels of pathogenic mtDNA, whereas functionally impaired cells with high mutational burden decline in competitiveness. Additional quality control mechanisms, including mitochondrial (**C**) fusion and (**D**) fission dynamics, safeguard mitochondrial integrity by complementing damaged mitochondria with healthy ones and selectively removing mutant mtDNA variants. **E**, Mitophagy, via the PINK1 (PTEN [Phosphatase and tensin homolog]-induced kinase 1)–Parkin pathway, further reduces heteroplasmy through the selective degradation of unhealthy mitochondria. Together, these processes preserve cellular bioenergetics and delay the onset of heteroplasmy-driven pathology in mitotic tissues.

Mitotic tissues are composed of cells that are capable of cell division and proliferation through mitosis. This facilitates tissue growth, renewal, and repair. In such tissues, several intrinsic mechanisms work synergistically to dilute the mutant mtDNA proportions and buffer heteroplasmy effects, limiting functional impact on tissue function (Figure 2).

One of the most fundamental mechanisms mitigating mitochondrial heteroplasmy in mitotic tissues is the process of vegetative segregation (Figure 2A) of the mtDNA.^23,45,66^ During mitosis, the mitochondria undergo mitochondrial fission, and the organelle divides between endoplasmic reticulum–constricted sites enriched in replicating mtDNA.^67^ Replication of mtDNA precedes division, generating new mtDNA, available across the mitochondrial network to be distributed among daughter cells in a random fashion.^45,68^ This stochastic segregation results in a random distribution of mutant and wild-type mtDNA, allowing some cell lineages to inherit relatively fewer mutant variants.^23^ Over successive generations of cell division, vegetative segregation can markedly reduce the average mitochondrial heteroplasmy in mitotic tissues. Beyond mere dilution due to replication, mitotic tissues also exercise clonal expansion (Figure 2B) and natural selection of healthy cells to preserve mitochondrial integrity.^69^ Cells harboring high loads of pathogenic mtDNA variants often suffer functional impairments, leading to reduced proliferation, making them less competitive. Over time, cell lineages with wild-type mtDNA and healthy mitochondrial function expand preferentially.^70^

In addition, mitotic tissues maintain mitochondrial health through intracellular selection and turnover mechanisms (Figure 2C through 2E), including mitochondrial fusion, fission, and mitophagy.^71^ Although they are not unique to proliferative cells only, but are also present in postmitotic tissues, their existence further decreases mitochondrial heteroplasmy. Mitophagy (Figure 2E) is the selective autophagic process for the removal of defective mitochondria,^72,73^ often characterized by high heteroplasmic levels of pathogenic mtDNA variants. This quality control mechanism occurs through the PINK1 (PTEN [Phosphatase and tensin homolog]-induced kinase 1)–Parkin pathway.^74–76^ on mitochondrial damage, PINK1 accumulates on the outer mitochondrial membrane and recruits Parkin. In turn, Parkin ubiquitinates several outer mitochondrial membrane proteins, tagging the organelle for degradation. Mitochondrial fission (Figure 2C) and fusion (Figure 2D) allow mutated and damaged mtDNA to be segregated or complemented by wild-type mtDNA, respectively.^77,78^ This further facilitates the bioenergetic homeostasis of the mitochondria, and ultimately of the cell.

Together, these mechanisms synergistically mitigate the pathogenic impact of mitochondrial heteroplasmy. However, their failure or insufficiencies can lead to clinical symptoms and disease.^77^ Nonepidermolytic palmoplantar keratoderma is a rare skin disorder that exemplifies this breakdown.^79,80^ Nonepidermolytic palmoplantar keratoderma is characterized by thickened skin of the palms and soles without epidermolysis. Although most nonepidermolytic palmoplantar keratoderma cases are linked to nuclear gene mutations,^81,82^ a subset is associated with the A7445G point mutation in the mtDNA.^79,80^ Patients harboring the mtDNA A7455G mutation develop nonepidermolytic palmoplantar keratoderma, which is also typically accompanied by sensorineural hearing loss.^79,80^

Overall, there are several mechanisms present in mitotic tissues to effectively reduce the mutational burden of mitochondrial heteroplasmy, although their failure, or absence in the case of postmitotic tissues, can lead to disease.

### Postmitotic Tissues

Unlike mitotic cells, postmitotic cells, such as neurons, myocytes, and cardiomyocytes, cannot proliferate and divide their mutation burden to dilute mitochondrial heteroplasmy. This may lead to a progressive accumulation of heteroplasmic mtDNA variants over time due to the multiple intrinsic and extrinsic factors that influence the accumulation of mutant mtDNA within cells (Figure 1).^83^ Mitotic tissues may reduce such variants over successive generations through processes including vegetative segregation that randomly distributes wild-type and pathogenic mitochondria across daughter cells, clonal expansion favoring the proliferation of lineages with lower levels of pathogenic mtDNA, and mitochondrial quality control mechanisms (Figure 2). To preserve mitochondrial respiratory function and OXPHOS capacity, postmitotic tissues heavily rely only on internal mitochondrial quality control mechanisms to manage the mutational burden, such as mitochondrial fission and fusion, as well as mitophagy. Specifically, Kandul et al^84^ observed a selective reduction of a heteroplasmic deletion in the mtDNA on activation of the PINK1-Parkin pathway in adult *Drosophila* muscle. However, the efficiency of these mitochondrial turnover mechanisms declines with age and cellular stress, leading to an increase in mitochondrial heteroplasmy over time.^85,86^ The interplay between inefficient turnover and high energy demand makes postmitotic tissues particularly susceptible to the clinical consequences of mitochondrial heteroplasmy.

Several well-characterized mitochondrial syndromes exemplify this principle. MELAS is one of the most common disorders associated with mitochondrial heteroplasmy. MELAS is primarily caused by mutations in the *MT-TL1* gene, primarily the m.3243A>G point mutation,^64,87^ although other mutations have also been reported.^88,89^ Clinical manifestation of MELAS usually occurs due to a moderate level of mtDNA heteroplasmy (typically 50%–60%) but is associated with severe bioenergetic defects (Table). Importantly, disease severity correlates with heteroplasmy burden, with lower levels resulting in a milder or different phenotype.^49–51,90^ Beyond MELAS, the same m.3243A>G point mutation in the *MT-TL1* gene can also give rise to maternally inherited diabetes and deafness.^52,53^ This syndrome is primarily characterized by diabetes and sensorineural hearing loss,^53,57,58^ generally associated with a mitochondrial heteroplasmy threshold ≥60% in skeletal muscle and more moderate levels in β cells (≈25%–70%; Table).^58^ This illustrates how tissue-specific mitochondrial heteroplasmy dynamics can affect clinical presentation, despite the presence of the same pathogenic mtDNA variant.

Several other mitochondrial disorders affecting postmitotic tissues highlight the impact of heteroplasmy. Myoclonic epilepsy with ragged-red fibers, typically linked to the m.8344A>G point mutation in the *MT-TK* gene (transfer RNA^Lys^), primarily affects skeletal muscle and the nervous system, producing myoclonus, epilepsy, ataxia, and ragged-red fibers (Table).^60,91^ Neuropathy, ataxia, and retinitis pigmentosa, most commonly caused by mutations in the *MT-ATP6* gene (mitochondrial ATP synthase membrane subunit 6), manifest in neurons and photoreceptors, leading to peripheral neuropathy, vision loss, and ataxia (Table).^92,93^ Interestingly, higher mutation burden in the *MT-ATP6* gene shifts the phenotype to Leigh syndrome, a severe infantile-onset neurodegenerative disease, causing progressive neurological decline and early mortality (Table).^93^ Next to point mutations, Kearns-Sayre syndrome is associated with large-scale mtDNA deletions, typically sporadic, that exist in a heteroplasmic state.^94–96^ Kearns-Sayre syndrome presents with chronic progressive external ophthalmoplegia and pigmentary retinopathy.^94–96^ In contrast to other metabolic disorders caused by point mutations, Kearns-Sayre syndrome–associated large-scale mtDNA deletions do not exhibit a clearly defined universal mitochondrial heteroplasmy threshold. Collectively, all these disorders illustrate how mitochondrial heteroplasmy drives clinical phenotypes in postmitotic tissues.

Although neuromuscular and neurological disorders have been extensively studied as consequences of mitochondrial heteroplasmy,^49–53,57,58,60,64,87–96^ the heart represents an equally energy-demanding organ that is particularly susceptible to mitochondrial damage^48,97^ and remains underexplored.

## Heart as an Underexplored Tissue

The heart is among the most metabolically active organs in the human body, with its ATP production and consumption rates far exceeding those of other tissues to sustain its continuous contractions. Cardiomyocytes are densely packed with mitochondria, which can occupy up to 25% to 30% of their cellular volume^98^ Therefore, OXPHOS plays a central role in cardiac physiology and function, producing over 90% of the ATP required for cardiomyocyte energy needs.^99^

This heavy reliance on mitochondrial energy production suggests that impairments in the mtDNA and subsequently impaired energy supply can have severe consequences in cardiac function. Supporting this idea, Yonova-Doing et al,^100^ in a recent population study encompassing 502 682 participants, identified numerous associations between mitochondrial single-nucleotide variants and complex traits, including cardiovascular disease phenotypes. Although these findings were primarily based on homoplasmic variation, the fact that common mitochondrial single-nucleotide variants can measurably influence cardiovascular outcomes highlights the sensitivity of the heart to mitochondrial genetic perturbations. Despite this, direct studies on mitochondrial impairment, and especially focusing on the heteroplasmy phenomenon in the heart, are surprisingly limited. As a result, most of the existing research has instead focused on mtDNA copy number, defined as the total number of mtDNA molecules within a cell, rather than systematically addressing mitochondrial heteroplasmy itself as a distinct variable. Large-scale genomic studies have identified both nuclear and mitochondrial genetic variants associated with copy number regulation,^101^ and although not necessarily causal, reduced mtDNA copy number has been associated with an elevated risk of cardiovascular disease and heart failure.^102–106^ One possible explanation is that changes in mtDNA copy number may, in some contexts, reflect cellular responses to mitochondrial genetic stress, including mitochondrial heteroplasmy. For example, mitophagy can preferentially target mitochondria carrying high loads of mutant mtDNA, thereby lowering the pathogenic threshold, hence reducing the overall abundance of mtDNA within a cell.^72,73^ However, existing studies remain largely associative and do not establish a direct causal relationship between mtDNA copy number and mitochondrial heteroplasmy.

Existing heteroplasmy data indicate that heteroplasmic mtDNA mutations contribute to late-onset cardiac dysfunction and risk of cardiovascular disease. Lechuga-Vieco et al^97^ engineered mice with divergent nonpathological mtDNA heteroplasmy, which developed severe (cardio-) muscular pathologies, including pulmonary hypertension, heart failure, muscle weakness, and premature death. This work highlighted that heteroplasmic mtDNA variants, typically regarded as nonpathogenic, can drive maladaptive outcomes in the heart. Clinical evidence aligns with these experimental findings. In MELAS and maternally inherited diabetes and deafness, heteroplasmic mutations in the *MT-TL1* gene not only cause neurological and muscular symptoms, but, in 1 of 3 patients, it also manifests as cardiac complications, such as hypertrophic cardiomyopathy and dilated cardiomyopathy,^54,55,107–110^, as well as conduction defects (Table).^107,111,56^

Similarly, heteroplasmic *MT-TK* mutations causing myoclonic epilepsy with ragged-red fibers have also been associated with hypertrophic/dilated cardiomyopathy ^112,113^ and an elevated risk of sudden cardiac death due to arrhythmias (Table).^112^ Notably, Leigh syndrome, associated with high heteroplasmy levels in *MT-ATP6*, can also present cardiac involvement,^114^ whereas neuropathy, ataxia, and retinitis pigmentosa, arising from the same mutation as Leigh syndrome but with a lower mutational burden, typically do not present any cardiac manifestations (Table).^59^ Cardiac involvement is also well recognized in Kearns-Sayre syndrome–associated large-scale mtDNA deletions, frequently presenting conduction abnormalities including arrhythmias and atrioventricular block (Table).^61–63^ Importantly, patients with mitochondrial disease who also develop cardiomyopathy have been observed to have lower survival rates than those without any cardiac involvement, highlighting the clinical impact of heteroplasmy-driven cardiac dysfunction.^115^ Collectively, these examples illustrate that cardiac manifestations are prevalent in patients with mitochondrial heteroplasmy-induced metabolic disease, and considering their association with reduced survival, expanding our understanding of mitochondrial heteroplasmy in cardiac disease is particularly important.

Together, these experimental and clinical observations raise the question of how heteroplasmic mtDNA mutations translate into cardiac dysfunction. Direct mechanistic studies linking mitochondrial heteroplasmy to cardiac pathology are limited; however, extensive work in cardiac mitochondrial biology provides important insight into how heteroplasmic mtDNA variants could progressively reshape cardiomyocyte biology. In the heart, accumulation of heteroplasmic mtDNA variants over time is likely to place sustained pressure on the OXPHOS system.^116^ Over time, increased heteroplasmic burden may also lead to the disruption of complex-specific electron flow that alters the redox balance and promotes electron leak, further increasing mitochondrial ROS production.^117^ Increased ROS production has been shown to promote the accumulation of additional pathogenic mtDNA variants,^27^ propagating a vicious cycle. This concept is in line with an earlier concept, according to which, also in animal models of heart failure with reduced ejection fraction (but without prior mtDNA defects), a vicious cycle of increased ROS production from complex I leads to mtDNA damage, which in turn promotes even more mitochondrial ROS.^118–120^

In cardiomyocytes, chronic mitochondrial oxidative stress not only damages the mtDNA and mitochondrial components but also destabilizes cytosolic calcium (Ca^2+^) and sodium (Na^+^) handling, for instance, by inhibition of sarcoplasmic reticulum Ca^2+^ ATPase activity and oxidation of RyR2 (ryanodine receptor 2) activity, together resulting in decreased sarcoplasmic reticulum Ca^2+^ load and spontaneous sarcoplasmic reticulum Ca^2+^ release events that can trigger arrhythmias.^121^ Furthermore, ROS can activate the CaMKII (Ca^2+^/calmodulin-dependent protein kinase II),^122^ which further contributes to not only RyR2 phosphorylation but also activation of the late Na^+^ current, which in turn increases cytosolic Na^+^ concentrations.^123^ These alterations together hamper mitochondrial Ca^2+^ accumulation and thereby, Krebs cycle activation, which oxidizes pyridine nucleotides required for ATP production at the ETC (nicotinamide adenine dinucleotide) and the antioxidative capacity (nicotinamide adenine dinucleotide phosphate), the latter increasing mitochondrial ROS emission.^124^ As a result, a vicious cycle of mitochondrial ROS and defective excitation-contraction coupling emerges that leads to further decline of cardiac function and increased risk of arrhythmias.^121^

As mitochondrial dysfunction accumulates, cardiomyocytes rely heavily on quality control pathways to preserve bioenergetic capacity. In postmitotic cells, impaired or insufficient intracellular turnover mechanisms allow dysfunctional mitochondria carrying high heteroplasmic burdens to persist.^85^ This reinforces a feed-forward cycle of energetic stress and oxidative damage. At the same time, persistent mitochondrial dysfunction can engage mitonuclear stress signaling, including activation of the mitochondrial unfolded protein response and altering nuclear gene expression that governs metabolism, mitochondrial quality control, and cellular survival.^125,126^ In more advanced stages, severely damaged mitochondria may release mtDNA into the cytosol, where it can act as a damage-associated molecular pattern and activate innate immune pathways, such as cGAS-STING (cGMP-AMP synthase–stimulator of interferon genes) signaling.^127,128^ Chronic low-grade inflammation driven by mitochondrial stress has been found to contribute to adverse myocardial remodeling and progression toward heart failure.^129^ Collectively, these interlinked processes suggest plausible mechanisms through which mitochondrial heteroplasmy could initiate a cascade of redox and stress responses that could further contribute to cardiomyocyte dysfunction and disease progression. This mechanistic framework is inferred from established knowledge in broader cardiac mitochondrial biology and heart failure. The proposed pathways warrant direct investigation in the human heart to determine whether mitochondrial heteroplasmy causally contributes to cardiac disease.

However, studying mitochondrial heteroplasmy in the heart presents major challenges. Most research focusing on heart disease relies on indirect measures of noncardiac tissue, such as blood (leukocytes).^107,108,112,130–132^ Although blood sampling is minimally invasive and practical, it does not reflect the mutational burden in energy-demanding postmitotic tissues, such as the heart.

## Tissue Accessibility and Reliance on Blood

One of the greatest barriers to advancing our understanding of mitochondrial heteroplasmy in clinically relevant tissues, such as the heart, is limited accessibility. Obtaining samples from cardiac tissues is highly invasive and often impractical and undesirable in patients. Consequently, researchers have historically relied on blood samples as a surrogate tissue because they are minimally invasive and therefore easy to collect.^107,108,112,130–132^

Blood stems from the hematopoietic system, which is a highly mitotic compartment.^133^ As a result, its nucleated components, such as leukocytes, undergo continuous cellular turnover in which heteroplasmic mtDNA dynamics are shaped by vegetative segregation, mitochondrial turnover, and purifying selection acting against pathogenic variants at the hematopoietic stem cell level, processes that collectively reduce the mutant mtDNA load in circulating blood cells over time.^23,45,134^ As a result, heteroplasmy levels measured in leukocytes often underestimate or fail to reflect the burden present in postmitotic and energy-demanding tissues, such as the heart and brain, where pathogenic consequences are most pronounced. Direct comparison shows that heteroplasmy levels differ significantly between postmitotic tissues and blood, specifically, in 56 individuals, skeletomuscular tissue consistently had 37% higher levels of heteroplasmy than blood.^135^ Adding further complexity, heteroplasmy levels in blood have been reported multiple times to decline with age,^135–137^ whereas the mutation load in skeletal muscle remained unchanged.^135^ These differences emphasize that while blood is an accessible proxy, it is not a reliable indicator of the mutational burden in postmitotic tissues. Therefore, blood-based measurements may obscure the true role of heteroplasmy in driving cardiac dysfunction. Consistent with this, the recent American Heart Association Scientific Statement on mitochondrial genetics in cardiovascular disease explicitly acknowledges that disease-causing mtDNA variants may be progressively lost in blood cells, particularly in adults, further underscoring that blood-based measurements provide an incomplete and potentially misleading picture of the true heteroplasmic burden in the heart.^19^

To overcome such limitations, alternative tissues and sampling strategies that may better approximate the heart are increasingly being explored. Skeletal muscle biopsies are often used as a proxy in cardiac research because of their high energetic demands and physiological similarities to cardiomyocytes in disease.^138–141^ However, emerging evidence suggests that the levels of heteroplasmy can differ substantially between cardiac and skeletal muscle,^142,143^ reflecting the heterogeneity of heteroplasmy across tissues. Even more relevant, endomyocardial biopsies (EMB), although invasive, remain the most accurate approach to directly measure heteroplasmy within the myocardium itself.^144^ EMB is considered the gold standard for diagnosing a range of cardiac conditions because it enables direct histological, molecular, and genetic evaluation of the heart, which cannot be reliably achieved through any of the current noninvasive proxies.^145–149^

Although the clinical application of EMB remains largely restricted to specific diagnostic indications or advanced research contexts, advances in molecular technologies have expanded for studying mitochondrial heteroplasmy. Next-generation sequencing platforms, particularly when applied with deep coverage, have been extensively applied to detect heteroplasmic variants.^150–153^ To ensure accuracy, specialized computational pipelines, such as mtDNA-Server, in combination with robust error-correction strategies, are essential for distinguishing true heteroplasmic variants from sequencing artifacts or nuclear mtDNA segments.^154^ In parallel, mtDNA analysis by rolling circle amplification and sequencing is emerging as a promising tool able to streamline library preparation for next-generation sequencing.^155^ By allowing high-throughput and sensitive detection of low-frequency heteroplasmic variants (1%), this method holds promise for large cohort studies where efficiency and precision are equally critical.^155^ Furthermore, long-read sequencing technologies now enable full-length mtDNA analysis and improved detection of large-scale deletions, structural rearrangements, and phasing of mtDNA variants, while reducing interference from or nuclear mtDNA segments.^156^ Most recently, single-cell mtDNA sequencing approaches, such as mitochondrial single-cell ATAC-sequencing have further expanded the ability to resolve heteroplasmy at cellular resolution, revealing substantial intratissue variability that is obscured in bulk sequencing approaches.^157,158^ These techniques may be particularly relevant for the myocardium, where cardiomyocytes and nonmyocyte populations may harbor distinct heteroplasmy profiles. Complementary targeted approaches, such as digital droplet polymerase chain reaction, have also emerged as highly sensitive quantitative methods.^159^ They could be utilized for validating low-frequency heteroplasmic mtDNA variants and accurately measuring mtDNA mutant loads in clinical and experimental settings.

Given the limitations of EMB in terms of invasiveness, patient-specific in vitro models are increasingly being considered as alternative approaches. Among these, human induced pluripotent stem cell–derived cardiomyocytes (hiPSC-CMs) provide a promising tool for studying mitochondrial heteroplasmy in a cardiac context.^160–163^ Somatic cells, such as skin fibroblasts, due to their intrinsic mechanisms to reduce heteroplasmy, naturally exhibit variable levels of mtDNA mutations. hiPSCs can be generated from patient-derived somatic cells, enabling the production of hiPSC lines with differing heteroplasmy from the same individual.^161,164^ Notably, these heteroplasmy levels are largely preserved during cardiac differentiation, enabling the study of load-dependent effects that correlate with clinical phenotypes.^160,163^ Specifically, Yokota et al^165^ reported that high levels of the m.3243A>G point mutation (>90%) in MELAS patient-derived hiPSC-CMs were associated with mitochondrial respiratory dysfunction and impaired cardiac lineage commitment. Lower levels of heteroplasmic burden (40%–90%) were linked to enhanced cardiogenic potential and normal contractility, comparable to the isogenic hiPSC-CMs (<40% heteroplasmy).^166^ These findings demonstrate that hiPSC-CMs could provide a renewable and physiologically relevant platform that allows for detailed mechanistic studies of heteroplasmic mtDNA mutations and their burden on cardiac function. Furthermore, they could serve as a preclinical model for evaluating therapeutic interventions, thereby complementing EMBs by offering accessibility and scalability without associated risks of invasive sampling.

Although hiPSC-CMs provide a valuable platform to study patient-specific mitochondrial heteroplasmy in human cardiomyocytes, several limitations remain. Most notably, hiPSC-CMs retain an immature phenotype characterized by glycolysis-dominant metabolism, underdeveloped mitochondrial architecture, and reduced electrophysiological and contractile maturity compared with adult cardiomyocytes.^167–169^ These features may influence mitochondrial function and potentially affect the interpretation of heteroplasmy-related phenotypes. Recent efforts to improve maturation, including long-term culture, electrical stimulation, fatty acid supplementation, and 3-dimensional engineered tissue systems, have shown promise in promoting adult-like phenotypes.^167–169^ However, the field is still evolving, and it remains unclear to what extent current maturation protocols influence heteroplasmy dynamics.

Methodological advances significantly enhance our ability to characterize cardiac heteroplasmy with greater precision, providing tools to bridge the gap between invasive sampling and meaningful biological insights.^166^

## Future Perspectives

Expanding our understanding of mitochondrial heteroplasmy in the heart carries significant implications for therapeutic development. Currently, emerging strategies aimed at manipulating mitochondrial heteroplasmy offer potential routes to reduce the burden of pathogenic mtDNA variants and restore cellular bioenergetics, shifting heteroplasmy back to a wild-type state.

Engineered mitochondrially targeted nucleases have been seen to selectively recognize and cleave pathogenic heteroplasmic mtDNA variants, leading to their degradation, allowing the wild-type variants to repopulate within the mitochondrial network. mtZFNs (mitochondrial-targeted zinc finger nucleases) have been shown to successfully shift m.8993T>G heteroplasmy in vitro,^170,171^ a point mutation most associated with neuropathy, ataxia, and retinitis pigmentosa and Leigh syndrome (Table), followed by rescue of mitochondrial function within the mtZFN-positive cells.^171^ In addition, mtRE (mitochondrial-directed restriction endonucleases)^172–174^ and mtTALENS (mitochondrial transcription activator–like effector nucleases)^175–177^ have been reported to selectively recognize and cleave mutant mtDNA in preclinical models. Adeno-associated virus vectors have been successfully used to deliver these nucleases to specific tissues, such as skeletal muscles and the heart.^172,178^ Notably, Bacman et al^172^ administered mito-ApaLI (mtRE), packaged within adeno-associated virus 9 vectors, in neonatal heteroplasmic mice harboring 2 distinct variants, only one of which was sensitive to ApaLI cleavage. The authors reported a consistent and robust shift in mtDNA heteroplasmy across the examined murine muscles, including the heart.^172^

Alongside mitochondrial nuclease-based approaches, mitochondrial base editing has emerged as an alternative strategy capable of directly converting pathogenic mtDNA variants without introducing double-strand breaks. Mitochondrial dddA-derived cytosine base editors were the first programmable systems shown to mediate C:G to T:A conversions within the mtDNA.^179^ This allowed the field to overcome the limitations of conventional CRISPR/Cas (clustered regularly interspaced short palindromic repeats/clustered regularly interspaced short palindromic repeat–associated) systems to efficiently target mitochondria due to guide RNA import limitations.^180^ Silva-Pinheiro et al^181^ delivered mitochondrial dddA-derived cytosine base editors systematically using cardiotropic adeno-associated virus 9 vectors. They demonstrated successful mtDNA editing in murine cardiac tissue, reaching 10% to 20% editing in adult and 20% to 30% editing in neonatal hearts, without changes in mtDNA copy number.^181^ Building on this platform, mtTALEDs (mitochondrial transcription activator–like effector–linked deaminases) have subsequently enabled T:A to C:G editing within the mtDNA.^182^ This substantially expanded the range of potentially targetable pathogenic mtDNA variants, including those causing MELAS and maternally inherited diabetes and deafness (m.3243A>G), myoclonic epilepsy with ragged-red fibers (m.8344A>G), neuropathy, ataxia, retinitis pigmentosa, and Leigh syndrome (m.8993T>G). Cho et al^182^ demonstrated that mtTALED achieved efficient adenine base editing across multiple human mitochondrial genes in vitro, with A to G conversion efficiencies ranging from 10% to 50%.^182^

In recent years, it has become increasingly recognized that mitochondria can be transferred between different cell types in vitro and in vivo.^183^ Such transfer is achieved either by tunneling nanotubules between cells, the release and capture of extracellular vesicles containing mitochondria as cargo, or also of naked mitochondria.^183^ The fate of mitochondria being captured into recipient cells is still unclear, because to function in the cellular context, they would need to evade endo(lyso)somes and their degradation therein.^183^

Nevertheless, motivated by these discoveries, horizontal mitochondrial transplantation (HMT) was established as a therapeutic avenue for enhancing mitochondrial quality in cardiovascular diseases. During HMT, mitochondria from donor cells are delivered to acceptor cells, with the mitochondria transplant sourced from the same (autologous HMT) or different (heterologous HMT) organism of the same species.^184^ In the cardiovascular system, this technique was particularly established by McCully et al,^185^ who reintroduced isolated mitochondria (often isolated from skeletal muscle) into the site of myocardial ischemia in rabbits to restore the tissue’s functionality. The approach was later translated into pediatric patients undergoing cardiac surgery with severe postischemic reperfusion cardiac shock via direct epicardial injection.^186,187^ Despite these promising observations, several fundamental mechanistic uncertainties must be acknowledged. Although HMT showed improvements in cardiac function in models of myocardial infarction and heart failure, currently, no studies have directly investigated its potential in shifting mitochondrial heteroplasmy in cardiovascular disease. The extracellular environment presents a formidable barrier to isolated mitochondria, as millimolar Ca^2+^ concentrations in both the interstitial space and bloodstream may trigger mitochondrial permeability transition pore opening, leading to organelle swelling and loss of membrane potential before any cellular uptake.^188^ Furthermore, the proportion of transplanted mitochondria that successfully enter cardiomyocytes in vivo remains poorly defined, and it is unclear whether uptake occurs at a scale sufficient to meaningfully alter cellular energetics or heteroplasmic load. Finally, the rapid functional improvements reported in some animal models and pediatric series occur within hours of injection, a timescale that raises questions about whether sufficient mitochondria have survived the extracellular environment and completed cellular uptake at the scale required to drive meaningful energetic recovery. Related to these uncertainties, as well as the observation that mitochondrial transfer between cells in nature is governed by extracellular vesicles carrying mitochondria or mitochondrial content, more recent approaches have progressively focused on delivering mitochondria in extracellular vesicles.^189^ Although intracellular uptake of transplanted mitochondria has been demonstrated in vitro^190^ and in murine cardiac models in vivo,^191^ the translational relevance of these findings to the human heart remains uncertain. The remaining questions concern not only whether uptake can occur but also whether incorporated mitochondria persist over clinically meaningful timeframes, functionally integrate into the host mitochondrial network, and contribute to the recipient cell’s energetic output or heteroplasmic landscape at a therapeutically relevant scale. Taken together, despite promising functional effects especially in the field of ischemia/reperfusion, the mechanisms of benefit provided by HMT are currently incompletely understood, and it is conceptually at least questionable whether HMT is a promising strategy to improve mitochondrial function in conditions with mitochondrial heteroplasmy, especially in the absence of any supporting evidence at this point.

Despite the conceptual appeal of both mtDNA editing approaches and HMT, important translational challenges also remain that are particularly relevant in the context of the human heart. Achieving efficient, homogenous delivery across the myocardium represents a shared barrier for all strategies. Specifically, most in vivo studies on nuclease-based approaches have relied on neonatal/murine models or striated muscle with high adeno-associated virus penetrance,^192,193^ whereas key questions persist regarding the efficiency of mitochondrial uptake by (adult) human cardiomyocytes in HMT,^189^ especially because clinical trials have been conducted only in pediatric patients.^186,187^ Immunologic compatibility also represents an important translational hurdle for all potential therapies. Notably, in mitochondrial nuclease-based and base-editing approaches, immune responses to viral vectors or enzyme editing components remain insufficiently understood. Adeno-associated virus delivery can activate both innate and adaptive immunity, including the complement system, cytokine release, neutralizing antibodies, and capsid-specific CD8^+^ T cells that clear transduced cells and have caused toxicities in multiple trials.^194–196^ Similarly, especially in heterologous HMT, mitigating immune responses remains an important challenge that needs to be addressed before its application in cardiac disease. In small-animal models and pediatric series, HMT administered directly to the heart did not provoke overt cytokine surges, allogenic reactivity, or damage-associated molecular pattern responses, suggesting low baseline immunogenicity.^185–187^ Nonetheless, in a murine heart-transplant model, donor-derived circulating mitochondria activated endothelial and dendritic cells, leading to accelerated allograft rejection.^197^ This suggests that extracellular mitochondria can behave as potent danger signals, inducing damage-associated molecular pattern responses. Therefore, the true immunogenicity profile of HMT remains unclear. However, it is worth considering that the same signals may not be exclusively detrimental. Vagnozzi et al^198^ demonstrated that intracardiac injection of nonviable cells and innate immune stimuli reproduced the functional cardiac improvement seen with stem cell therapy in a mouse model of ischemia-reperfusion injury, implicating an acute macrophage-driven wound-healing response rather than cellular engraftment as the principal mechanism of benefit. Extracellular mitochondria, which carry potent damage-associated molecular pattern signals including mtDNA, cardiolipin, and formyl peptides, may similarly elicit a transient innate immune response that contributes to the functional improvements reported in HMT studies, independently of durable mitochondrial engraftment. This alternative mechanism does not diminish the therapeutic interest in HMT but underscores that the basis of its reported benefits remains mechanistically unresolved and warrants dedicated investigation. Furthermore, mitochondria have limited mtDNA repair capabilities,^28–31^ raising concerns that nuclease-induced cleavage could lead to mtDNA depletion rather than controlled heteroplasmy shifting. Although base-editing approaches avoid double-strand cleavage and, therefore, reduce the risk of extensive mtDNA depletion, both mitochondrial dddA-derived cytosine base editors and mtTALEDs have been reported to induce unintended mtDNA edits.^199^ Therefore, dosing and specificity of both nuclease-based and base-editing approaches will need to be tightly regulated. Moreover, there is currently no direct evidence that HMT can durably alter mitochondrial heteroplasmy levels in the heart. As such, it may primarily modulate acute mitochondrial function rather than reprogram the underlying mitochondrial genetic landscape, restoring ATP production and respiration.^185^ Together, these considerations highlight that while both approaches represent powerful tools, substantial optimizations and safety evaluations will be required before they can be translated into effective therapies for heteroplasmy-driven cardiac disease.

Looking ahead, these insights also pave the way for personalized medicine in mitochondrial cardiology. Because heteroplasmy levels vary widely between patients and tissues,^45,135,142,143^ integrating heteroplasmy assessment into risk stratification frameworks could transform the management of patients with heteroplasmic mtDNA mutations associated with cardiac manifestations.^200^ Quantifying heteroplasmy levels may help identify individuals at higher risk of developing cardiomyopathy, arrhythmias, or heart failure,^201^ enabling earlier surveillance, and intervention tailored to the specific heteroplasmic landscape of each individual.

## Conclusions

Altogether, despite significant advances in mitochondrial diseases, the role of mitochondrial heteroplasmy in the heart remains insufficiently characterized because much of our current knowledge derives from surrogate tissues, such as blood. This leaves critical gaps in our understanding of how heteroplasmic mtDNA variants directly influence cardiac physiology and disease. Emerging therapeutic approaches demonstrate the feasibility of shifting heteroplasmy for restoring mitochondrial function, underscoring the potential clinical translation. Bridging these knowledge gaps will be essential for advancing both mechanistic insight and therapeutic innovation in mitochondrial cardiology.

## Article Information

### Disclosures

None.

## References

[1] Archibald JM. Endosymbiosis and eukaryotic cell evolution. Curr Biol. 2015;25:R911–R921. doi: 10.1016/j.cub.2015.07.055 26439354 10.1016/j.cub.2015.07.055

[2] Zimorski V Ku C Martin WF Gould SB. Endosymbiotic theory for organelle origins. Curr Opin Microbiol. 2014;22:38–48. doi: 10.1016/j.mib.2014.09.008 25306530 10.1016/j.mib.2014.09.008

[3] Anderson S Bankier AT Barrell BG de Bruijn MHL Coulson AR Drouin J Eperon IC Nierlich DP Roe BA Sanger F. Sequence and organization of the human mitochondrial genome. Nature. 1981;290:457–465. doi: 10.1038/290457a0 7219534 10.1038/290457a0

[4] Roger AJ Muñoz-Gómez SA Kamikawa R. The origin and diversification of mitochondria. Curr Biol. 2017;27:R1177–R1192. doi: 10.1016/j.cub.2017.09.015 29112874 10.1016/j.cub.2017.09.015

[5] Bonawitz ND Clayton DA Shadel GS. Initiation and beyond: multiple functions of the human mitochondrial transcription machinery. Mol Cell. 2006;24:813–825. doi: 10.1016/j.molcel.2006.11.024 17189185 10.1016/j.molcel.2006.11.024

[6] Wang L Zhou X Lu T. Role of mitochondria in physiological activities, diseases, and therapy. Mol Biomed. 2025;6:42. doi: 10.1186/s43556-025-00284-5 40536597 10.1186/s43556-025-00284-5 PMC12179052

[7] Kohler A Barrientos A Fontanesi F Ott M. The functional significance of mitochondrial respiratory chain supercomplexes. EMBO Rep. 2023;24:e57092. doi: 10.15252/embr.202357092 37828827 10.15252/embr.202357092 PMC10626428

[8] Kremer LS Rehling P. Coordinating mitochondrial translation with assembly of the OXPHOS complexes. Hum Mol Genet. 2024;33:R47–R52. doi: 10.1093/hmg/ddae025 38779773 10.1093/hmg/ddae025 PMC11112383

[9] Jang S Javadov S. Elucidating the contribution of ETC complexes I and II to the respirasome formation in cardiac mitochondria. Sci Rep. 2018;8:17732. doi: 10.1038/s41598-018-36040-9 30531981 10.1038/s41598-018-36040-9 PMC6286307

[10] Viscomi C Zeviani M. Strategies for fighting mitochondrial diseases. J Intern Med. 2020;287:665–684. doi: 10.1111/joim.13046 32100338 10.1111/joim.13046

[11] Smeitink J. Nuclear genes of human complex I of the mitochondrial electron transport chain: state of the art. Hum Mol Genet. 1998;7:1573–1579. doi: 10.1093/hmg/7.10.1573 9735378 10.1093/hmg/7.10.1573

[12] Formosa LE Dibley MG Stroud DA Ryan MT. Building a complex complex: assembly of mitochondrial respiratory chain complex I. Semin Cell Dev Biol. 2018;76:154–162. doi: 10.1016/j.semcdb.2017.08.011 28797839 10.1016/j.semcdb.2017.08.011

[13] Alston CL Rocha MC Lax NZ Turnbull DM Taylor RW. The genetics and pathology of mitochondrial disease. J Pathol. 2017;241:236–250. doi: 10.1002/path.4809 27659608 10.1002/path.4809 PMC5215404

[14] Quirós PM Mottis A Auwerx J. Mitonuclear communication in homeostasis and stress. Nat Rev Mol Cell Biol. 2016;17:213–226. doi: 10.1038/nrm.2016.23 26956194 10.1038/nrm.2016.23

[15] Stewart JB Chinnery PF. Extreme heterogeneity of human mitochondrial DNA from organelles to populations. Nat Rev Genet. 2021;22:106–118. doi: 10.1038/s41576-020-00284-x 32989265 10.1038/s41576-020-00284-x

[16] Radzvilavicius AL Hadjivasiliou Z Pomiankowski A Lane N. Selection for mitochondrial quality drives evolution of the germline. PLoS Biol. 2016;14:e2000410. doi: 10.1371/journal.pbio.2000410 27997535 10.1371/journal.pbio.2000410 PMC5172535

[17] Nissanka N Moraes CT. Mitochondrial DNA heteroplasmy in disease and targeted nuclease-based therapeutic approaches. EMBO Rep. 2020;21:e49612. doi: 10.15252/embr.201949612 32073748 10.15252/embr.201949612 PMC7054667

[18] Sciacco M Bonilla E Schon EA DiMauro S Moraes CT. Distribution of wild-type and common deletion forms of mtDNA in normal and respiration-deficient muscle fibers from patients with mitochondrial myopathy. Hum Mol Genet. 1994;3:13–19. doi: 10.1093/hmg/3.1.13 8162014 10.1093/hmg/3.1.13

[19] Fetterman JL Chinnery PF McClellan R Wallace DC Suomalainen A Ojala T Lewis SC Ballinger SW; Council on Peripheral Vascular Disease. Mitochondrial genetics in cardiovascular health and disease: a scientific statement from the American Heart Association. Circulation. 2026;153:e42–e68. doi: 10.1161/cir.0000000000001393 41328540 10.1161/CIR.0000000000001393

[20] Lee W Zamudio-Ochoa A Buchel G Podlesniy P Marti Gutierrez N Puigròs M Calderon A Tang HY Li L Mikhalchenko A. Molecular basis for maternal inheritance of human mitochondrial DNA. Nat Genet. 2023;55:1632–1639. doi: 10.1038/s41588-023-01505-9 37723262 10.1038/s41588-023-01505-9 PMC10763495

[21] Politi Y Gal L Kalifa Y Ravid L Elazar Z Arama E. Paternal mitochondrial destruction after fertilization is mediated by a common endocytic and autophagic pathway in *Drosophila*. Dev Cell. 2014;29:305–320. doi: 10.1016/j.devcel.2014.04.005 24823375 10.1016/j.devcel.2014.04.005

[22] Zhang H Burr SP Chinnery PF. The mitochondrial DNA genetic bottleneck: inheritance and beyond. Essays Biochem. 2018;62:225–234. doi: 10.1042/EBC20170096 29880721 10.1042/EBC20170096

[23] Stewart JB Chinnery PF. The dynamics of mitochondrial DNA heteroplasmy: implications for human health and disease. Nat Rev Genet. 2015;16:530–542. doi: 10.1038/nrg3966 26281784 10.1038/nrg3966

[24] Feric M Demarest TG Tian J Croteau DL Bohr VA Misteli T. Self-assembly of multi-component mitochondrial nucleoids via phase separation. EMBO J. 2021;40:e107165. doi: 10.15252/embj.2020107165 33619770 10.15252/embj.2020107165 PMC7957436

[25] Huh H Shen J Ajjugal Y Ramachandran A Patel SS Lee SH. Sequence-specific dynamic DNA bending explains mitochondrial TFAM’s dual role in DNA packaging and transcription initiation. Nat Commun. 2024;15:5446. doi: 10.1038/s41467-024-49728-6 38937458 10.1038/s41467-024-49728-6 PMC11211510

[26] Hahn A Zuryn S. Mitochondrial genome (mtDNA) mutations that generate reactive oxygen species. Antioxidants (Basel). 2019;8:392. doi: 10.3390/antiox8090392 31514455 10.3390/antiox8090392 PMC6769445

[27] Quan Y Xin Y Tian G Zhou J Liu X. Mitochondrial ROS-modulated mtDNA: a potential target for cardiac aging. Oxid Med Cell Longev. 2020;2020:9423593–9423511. doi: 10.1155/2020/9423593 32308810 10.1155/2020/9423593 PMC7139858

[28] Allkanjari K Baldock RA. Beyond base excision repair: an evolving picture of mitochondrial DNA repair. Biosci Rep. 2021;41:BSR20211320. doi: 10.1042/BSR20211320 34608928 10.1042/BSR20211320 PMC8527207

[29] Prakash A Doublié S. Base excision repair in the mitochondria. J Cell Biochem. 2015;116:1490–1499. doi: 10.1002/jcb.25103 25754732 10.1002/jcb.25103 PMC4546830

[30] Tadi SK Sebastian R Dahal S Babu RK Choudhary B Raghavan SC. Microhomology-mediated end joining is the principal mediator of double-strand break repair during mitochondrial DNA lesions. Mol Biol Cell. 2016;27:223–235. doi: 10.1091/mbc.E15-05-0260 26609070 10.1091/mbc.E15-05-0260 PMC4713127

[31] Dahal S Dubey S Raghavan SC. Homologous recombination-mediated repair of DNA double-strand breaks operates in mammalian mitochondria. Cell Mol Life Sci. 2018;75:1641–1655. doi: 10.1007/s00018-017-2702-y 29116362 10.1007/s00018-017-2702-y PMC11105789

[32] Falkenberg M. Mitochondrial DNA replication in mammalian cells: overview of the pathway. Essays Biochem. 2018;62:287–296. doi: 10.1042/EBC20170100 29880722 10.1042/EBC20170100 PMC6056714

[33] Anderson AP Luo X Russell W Yin YW. Oxidative damage diminishes mitochondrial DNA polymerase replication fidelity. Nucleic Acids Res. 2020;48:817–829. doi: 10.1093/nar/gkz1018 31799610 10.1093/nar/gkz1018 PMC6954441

[34] Bratic A Kauppila TES Macao B Grönke S Siibak T Stewart JB Baggio F Dols J Partridge L Falkenberg M. Complementation between polymerase- and exonuclease-deficient mitochondrial DNA polymerase mutants in genomically engineered flies. Nat Commun. 2015;6:8808. doi: 10.1038/ncomms9808 26554610 10.1038/ncomms9808 PMC4773887

[35] Falkenberg M Larsson NG Gustafsson CM. Replication and transcription of human mitochondrial DNA. Annu Rev Biochem. 2024;93:47–77. doi: 10.1146/annurev-biochem-052621-092014 38594940 10.1146/annurev-biochem-052621-092014

[36] Larsson NG. Somatic mitochondrial DNA mutations in mammalian aging. Annu Rev Biochem. 2010;79:683–706. doi: 10.1146/annurev-biochem-060408-093701 20350166 10.1146/annurev-biochem-060408-093701

[37] Pinto M Moraes CT. Mechanisms linking mtDNA damage and aging. Free Radic Biol Med. 2015;85:250–258. doi: 10.1016/j.freeradbiomed.2015.05.005 25979659 10.1016/j.freeradbiomed.2015.05.005 PMC4508218

[38] Klein HU Trumpff C Yang HS Lee AJ Picard M Bennett DA De Jager PL. Characterization of mitochondrial DNA quantity and quality in the human aged and Alzheimer’s disease brain. Mol Neurodegener. 2021;16:75. doi: 10.1186/s13024-021-00495-8 34742335 10.1186/s13024-021-00495-8 PMC8572491

[39] Trifunovic A Wredenberg A Falkenberg M Spelbrink JN Rovio AT Bruder CE Bohlooly-Y M Gidlöf S Oldfors A Wibom R. Premature ageing in mice expressing defective mitochondrial DNA polymerase. Nature. 2004;429:417–423. doi: 10.1038/nature02517 15164064 10.1038/nature02517

[40] Leuthner TC Meyer JN. Mitochondrial DNA mutagenesis: feature of and biomarker for environmental exposures and aging. Curr Environ Health Rep. 2021;8:294–308. doi: 10.1007/s40572-021-00329-1 34761353 10.1007/s40572-021-00329-1 PMC8826492

[41] Fontana GA Singh NK Rotankova N Eichelberg A Di Filippo M MacArthur MR Heldmaier S Wandrey F Beer HD Sturla SJ. UVA irradiation promotes ROS-mediated formation of the common deletion in mitochondrial DNA. Life (Basel). 2026;16:577. doi: 10.3390/life16040577 42073386 10.3390/life16040577 PMC13117788

[42] Reddam A McLarnan S Kupsco A. Environmental chemical exposures and mitochondrial dysfunction: a review of recent literature. Curr Environ Health Rep. 2022;9:631–649. doi: 10.1007/s40572-022-00371-7 35902457 10.1007/s40572-022-00371-7 PMC9729331

[43] Bhutani V Varzideh F Wilson S Kansakar U Jankauskas S Santulli G. Doxorubicin-induced cardiotoxicity: a comprehensive update. J Cardiovasc Dev Dis. 2025;12:207. doi: 10.3390/jcdd12060207 40558642 10.3390/jcdd12060207 PMC12193598

[44] Qing G Huang C Pei J Peng B. Alteration of cardiac energetics and mitochondrial function in doxorubicin‑induced cardiotoxicity: molecular mechanism and prospective implications (review). Int J Mol Med. 2025;56:183. doi: 10.3892/ijmm.2025.5624 40910252 10.3892/ijmm.2025.5624 PMC12425352

[45] Burr SP Chinnery PF. Origins of tissue and cell-type specificity in mitochondrial DNA (mtDNA) disease. Hum Mol Genet. 2024;33:R3–R11. doi: 10.1093/hmg/ddae059 38779777 10.1093/hmg/ddae059 PMC11112380

[46] Cantó-Santos J Grau-Junyent JM Garrabou G. The impact of mitochondrial deficiencies in neuromuscular diseases. Antioxidants (Basel). 2020;9:964. doi: 10.3390/antiox9100964 33050147 10.3390/antiox9100964 PMC7600520

[47] Sansbury BE Jones SP Riggs DW Darley-Usmar VM Hill BG. Bioenergetic function in cardiovascular cells: the importance of the reserve capacity and its biological regulation. Chem Biol Interact. 2011;191:288–295. doi: 10.1016/j.cbi.2010.12.002 21147079 10.1016/j.cbi.2010.12.002 PMC3090710

[48] Elorza AA Soffia JP. mtDNA heteroplasmy at the core of aging-associated heart failure. An integrative view of OXPHOS and mitochondrial life cycle in cardiac mitochondrial physiology. Front Cell Dev Biol. 2021;9:625020. doi: 10.3389/fcell.2021.625020 33692999 10.3389/fcell.2021.625020 PMC7937615

[49] Lin DS Huang YW Ho CS Huang TS Lee TH Wu TY Huang ZD Wang TJ. Impact of mitochondrial A3243G heteroplasmy on mitochondrial bioenergetics and dynamics of directly reprogrammed MELAS neurons. Cells. 2022;12:15. doi: 10.3390/cells12010015 36611807 10.3390/cells12010015 PMC9818214

[50] Chae HW Na JH Kim HS Lee YM. Mitochondrial diabetes and mitochondrial DNA mutation load in MELAS syndrome. Eur J Endocrinol. 2020;183:505–512. doi: 10.1530/EJE-20-0189 33107434 10.1530/EJE-20-0189

[51] McMillan RP Stewart S Budnick JA Caswell CC Hulver MW Mukherjee K Srivastava S. Quantitative variation in m.3243A > G mutation produce discrete changes in energy metabolism. Sci Rep. 2019;9:5752. doi: 10.1038/s41598-019-42262-2 30962477 10.1038/s41598-019-42262-2 PMC6453956

[52] de Laat P Koene S den van Heuvel LPWJ Rodenburg RJT Janssen MCH Smeitink JAM. Clinical features and heteroplasmy in blood, urine and saliva in 34 Dutch families carrying the m.3243A > G mutation. J Inher Metab Dis. 2012;35:1059–1069. doi: 10.1007/s10545-012-9465-2 22403016 10.1007/s10545-012-9465-2 PMC3470685

[53] Ng N Sanchez-Lechuga B McCarrick C Mangan C Burke M Ioana JA Gavin C O’Byrne R O’Byrne JJ Byrne MM. Mitochondrial heteroplasmy-phenotype correlation and response to glucose lowering therapy in subjects with m.3243A>G mutations. Diabetes Metab. 2025;51:101678. doi: 10.1016/j.diabet.2025.101678 40541739 10.1016/j.diabet.2025.101678

[54] Majamaa-Voltti K Peuhkurinen K Kortelainen ML Hassinen IE Majamaa K. Cardiac abnormalities in patients with mitochondrial DNA mutation 3243A>G. BMC Cardiovasc Disord. 2002;2:12. doi: 10.1186/1471-2261-2-12 12150714 10.1186/1471-2261-2-12 PMC119851

[55] Azevedo O Vilarinho L Almeida F Ferreira F Guardado J Ferreira M Lourenço A Medeiros R Almeida J. Cardiomyopathy and kidney disease in a patient with maternally inherited diabetes and deafness caused by the 3243A>G mutation of mitochondrial DNA. Cardiology. 2010;115:71–74. doi: 10.1159/000252811 19864902 10.1159/000252811

[56] Suzuki S Oka Y Kadowaki T Kanatsuka A Kuzuya T Kobayashi M Sanke T Seino Y Nanjo K; Research Committee or Specific Types of Diabetes Mellitus with Gene Mutations of the Japan Diabetes Society. Clinical features of diabetes mellitus with the mitochondrial DNA 3243 (A-G) mutation in Japanese: maternal inheritance and mitochondria-related complications. Diabetes Res Clin Pract. 2003;59:207–217. doi: 10.1016/s0168-8227(02)00246-2 12590018 10.1016/s0168-8227(02)00246-2

[57] Duran J Martinez A Adler E. Cardiovascular manifestations of mitochondrial disease. Biology (Basel). 2019;8:34. doi: 10.3390/biology8020034 31083569 10.3390/biology8020034 PMC6628328

[58] Chanoine JP Thompson DM Lehman A. Diabetes associated with maternally inherited diabetes and deafness (MIDD): from pathogenic variant to phenotype. Diabetes. 2025;74:153–163. doi: 10.2337/db24-0515 39556456 10.2337/db24-0515 PMC11755681

[59] Limongelli G Tome-Esteban M Dejthevaporn C Rahman S Hanna MG Elliott PM. Prevalence and natural history of heart disease in adults with primary mitochondrial respiratory chain disease. Eur J Heart Fail. 2010;12:114–121. doi: 10.1093/eurjhf/hfp186 20083621 10.1093/eurjhf/hfp186

[60] Lombès A Diaz C Romero NB Ziegler F Fardeau M. Analysis of the tissue distribution and inheritance of heteroplasmic mitochondrial DNA point mutation by denaturing gradient gel electrophoresis in MERRF syndrome. Neuromuscul Disord. 1992;2:323–330. doi: 10.1016/s0960-8966(06)80003-9 1300181 10.1016/s0960-8966(06)80003-9

[61] Kabunga P Lau AK Phan K Puranik R Liang C Davis RL Sue CM Sy RW. Systematic review of cardiac electrical disease in Kearns-Sayre syndrome and mitochondrial cytopathy. Int J Cardiol. 2015;181:303–310. doi: 10.1016/j.ijcard.2014.12.038 25540845 10.1016/j.ijcard.2014.12.038

[62] Chawla S Coku J Forbes T Kannan S. Kearns-Sayre syndrome presenting as complete heart block. Pediatr Cardiol. 2008;29:659–662. doi: 10.1007/s00246-007-9040-z 17763890 10.1007/s00246-007-9040-z

[63] van Beynum I Morava E Taher M Rodenburg RJ Karteszi J Toth K Szabados E. Cardiac arrest in Kearns–Sayre syndrome. JIMD Rep. 2011;2:7–10. doi: 10.1007/8904_2011_32 23430846 10.1007/8904_2011_32 PMC3509832

[64] Goto Y Nonaka I Horai S. A mutation in the tRNA(Leu)(UUR) gene associated with the MELAS subgroup of mitochondrial encephalomyopathies. Nature. 1990;348:651–653. doi: 10.1038/348651a0 2102678 10.1038/348651a0

[65] Wang YX Le WD. Progress in diagnosing mitochondrial myopathy, encephalopathy, lactic acidosis, and stroke-like episodes. Chin Med J (Engl). 2015;128:1820–1825. doi: 10.4103/0366-6999.159360 26112726 10.4103/0366-6999.159360 PMC4733719

[66] Yan C Duanmu X Zeng L Liu B Song Z. Mitochondrial DNA: distribution, mutations, and elimination. Cells. 2019;8:379. doi: 10.3390/cells8040379 31027297 10.3390/cells8040379 PMC6523345

[67] Lewis SC Uchiyama LF Nunnari J. ER-mitochondria contacts couple mtDNA synthesis with mitochondrial division in human cells. Science. 2016;353:aaf5549. doi: 10.1126/science.aaf5549 27418514 10.1126/science.aaf5549 PMC5554545

[68] Wonnapinij P Chinnery PF Samuels DC. The distribution of mitochondrial DNA heteroplasmy due to random genetic drift. Am J Hum Genet. 2008;83:582–593. doi: 10.1016/j.ajhg.2008.10.007 18976726 10.1016/j.ajhg.2008.10.007 PMC2668051

[69] Wang Z Li Z Liu H Yang C Li X. Mitochondrial clonal mosaicism encodes a biphasic molecular clock of aging. Nat Aging. 2025;5:1637–1651. doi: 10.1038/s43587-025-00890-6 40425806 10.1038/s43587-025-00890-6 PMC12350168

[70] Lawless C Greaves L Reeve AK Turnbull DM Vincent AE. The rise and rise of mitochondrial DNA mutations. Open Biol. 2020;10:200061. doi: 10.1098/rsob.200061 32428418 10.1098/rsob.200061 PMC7276526

[71] Onishi M Yamano K Sato M Matsuda N Okamoto K. Molecular mechanisms and physiological functions of mitophagy. EMBO J. 2021;40:e104705. doi: 10.15252/embj.2020104705 33438778 10.15252/embj.2020104705 PMC7849173

[72] Ng MYW Wai T Simonsen A. Quality control of the mitochondrion. Dev Cell. 2021;56:881–905. doi: 10.1016/j.devcel.2021.02.009 33662258 10.1016/j.devcel.2021.02.009

[73] Pickles S Vigié P Youle RJ. Mitophagy and quality control mechanisms in mitochondrial maintenance. Curr Biol. 2018;28:R170–R185. doi: 10.1016/j.cub.2018.01.004 29462587 10.1016/j.cub.2018.01.004 PMC7255410

[74] Lin Q Li S Jiang N Shao X Zhang M Jin H Zhang Z Shen J Zhou Y Zhou W. PINK1-parkin pathway of mitophagy protects against contrast-induced acute kidney injury via decreasing mitochondrial ROS and NLRP3 inflammasome activation. Redox Biol. 2019;26:101254. doi: 10.1016/j.redox.2019.101254 31229841 10.1016/j.redox.2019.101254 PMC6597739

[75] Eiyama A Okamoto K. PINK1/Parkin-mediated mitophagy in mammalian cells. Curr Opin Cell Biol. 2015;33:95–101. doi: 10.1016/j.ceb.2015.01.002 25697963 10.1016/j.ceb.2015.01.002

[76] Youle RJ Narendra DP. Mechanisms of mitophagy. Nat Rev Mol Cell Biol. 2011;12:9–14. doi: 10.1038/nrm3028 21179058 10.1038/nrm3028 PMC4780047

[77] Wang S Long H Hou L Feng B Ma Z Wu Y Zeng Y Cai J Zhang DW Zhao G. The mitophagy pathway and its implications in human diseases. Signal Transduct Target Ther. 2023;8:304. doi: 10.1038/s41392-023-01503-7 37582956 10.1038/s41392-023-01503-7 PMC10427715

[78] Lechado Terradas A Zittlau KI Macek B Fraiberg M Elazar Z Kahle PJ. Regulation of mitochondrial cargo-selective autophagy by posttranslational modifications. J Biol Chem. 2021;297:101339. doi: 10.1016/j.jbc.2021.101339 34688664 10.1016/j.jbc.2021.101339 PMC8591368

[79] Martin L Toutain A Guillen C Haftek M Machet MC Toledano C Arbeille B Lorette G Rötig A Vaillant L. Inherited palmoplantar keratoderma and sensorineural deafness associated with A7445G point mutation in the mitochondrial genome. Br J Dermatol. 2000;143:876–883. doi: 10.1046/j.1365-2133.2000.03797.x 11069477 10.1046/j.1365-2133.2000.03797.x

[80] Caria H Matos T Oliveira‐Soares R Santos A Galhardo I Soares‐Almeida L Dias O Andrea M Correia C Fialho G. A7445G mtDNA mutation present in a Portuguese family exhibiting hereditary deafness and palmoplantar keratoderma. J Eur Acad Dermatol Venereol. 2005;19:455–458. doi: 10.1111/j.1468-3083.2005.01087.x 15987292 10.1111/j.1468-3083.2005.01087.x

[81] Kelsell DP Stevens HP Ratnavel R Bryant SP Bishop DT Leigh IM Spurr NK. Genetic linkage studies in non-epidermolytic palmoplantar keratoderma: evidence for heterogeneity. Hum Mol Genet. 1995;4:1021–1025. doi: 10.1093/hmg/4.6.1021 7544664 10.1093/hmg/4.6.1021

[82] del Caño LR South AP O’Toole EA Kelsell DP Blaydon DC. A role for aquaporin-5 variants in regulation of the actin cytoskeleton in non-epidermolytic palmoplantar keratoderma. J Invest Dermatol. 2024;144:2092–2096. doi: 10.1016/j.jid.2024.02.028 38527693 10.1016/j.jid.2024.02.028

[83] Chiaratti MR Chinnery PF. Modulating mitochondrial DNA mutations: factors shaping heteroplasmy in the germ line and somatic cells. Pharmacol Res. 2022;185:106466. doi: 10.1016/j.phrs.2022.106466 36174964 10.1016/j.phrs.2022.106466

[84] Kandul NP Zhang T Hay BA Guo M. Selective removal of deletion-bearing mitochondrial DNA in heteroplasmic *Drosophila*. Nat Commun. 2016;7:13100. doi: 10.1038/ncomms13100 27841259 10.1038/ncomms13100 PMC5114534

[85] Duan M Tu J Lu Z. Recent advances in detecting mitochondrial DNA heteroplasmic variations. Molecules. 2018;23:323. doi: 10.3390/molecules23020323 29401641 10.3390/molecules23020323 PMC6017848

[86] Ye K Lu J Ma F Keinan A Gu Z. Extensive pathogenicity of mitochondrial heteroplasmy in healthy human individuals. Proc Natl Acad Sci USA. 2014;111:10654–10659. doi: 10.1073/pnas.1403521111 25002485 10.1073/pnas.1403521111 PMC4115537

[87] Goto Y Horai S Matsuoka T Koga Y Nihei K Kobayashi M Nonaka I. Mitochondrial myopathy, encephalopathy, lactic acidosis, and stroke-like episodes (MELAS): a correlative study of the clinical features and mitochondrial DNA mutation. Neurology. 1992;42:545–550. doi: 10.1212/wnl.42.3.545 1549215 10.1212/wnl.42.3.545

[88] Tarnopolsky MA Maguire J Myint T Applegarth D Robinson BH. Clinical, physiological, and histological features in a kindred with the T3271C melas mutation. Muscle Nerve. 1998;21:25–33. doi: 10.1002/(sici)1097-4598(199801)21:1<25::aid-mus4>3.0.co;2-i 9427220 10.1002/(sici)1097-4598(199801)21:1<25::aid-mus4>3.0.co;2-i

[89] Ikeda T Osaka H Shimbo H Tajika M Yamazaki M Ueda A Murayama K Yamagata T. Mitochondrial DNA 3243A>T mutation in a patient with MELAS syndrome. Hum Genome Var. 2018;5:25. doi: 10.1038/s41439-018-0026-6 30210801 10.1038/s41439-018-0026-6 PMC6123423

[90] Krylova T Itkis Y Tsygankova P Chistol D Lyamzaev K Tabakov V Mikhaylova S Nikitina N Rudenskaya G Murtazina A. Exploring the phenotypic heterogeneity and bioenergetic profile of the m.13513G>A mtDNA substitution: a heteroplasmy perspective. Int J Mol Sci. 2025;26:4565. doi: 10.3390/ijms26104565 40429710 10.3390/ijms26104565 PMC12111569

[91] Tanno Y Yoneda M Nonaka I Tanaka K Miyatake T Tsuji S. Quantitation of mitochondrial DNA carrying tRNALys mutation in MERRF patients. Biochem Biophys Res Commun. 1991;179:880–885. doi: 10.1016/0006-291x(91)91900-w 1910341 10.1016/0006-291x(91)91900-w

[92] Liu G Shen X Sun Y Lv Q Li Y Du A. Heteroplasmy and phenotype spectrum of the mitochondrial tRNALeu (UUR) gene m.3243A>G mutation in seven Han Chinese families. J Neurol Sci. 2020;408:116562. doi: 10.1016/j.jns.2019.116562 31722256 10.1016/j.jns.2019.116562

[93] Kara B Arikan M Maraş H Abaci N Çakiris A Üstek D. Whole mitochondrial genome analysis of a family with NARP/MILS caused by m.8993T>C mutation in the MT-ATP6 gene. Mol Genet Metab. 2012;107:389–393. doi: 10.1016/j.ymgme.2012.06.013 22819295 10.1016/j.ymgme.2012.06.013

[94] Grigalionienė K Burnytė B Balkelienė D Ambrozaitytė L Utkus A. Kearns-Sayre syndrome case. Novel 5,9 kb mtDNA deletion. Mol Genet Genomic Med. 2023;11:e2059. doi: 10.1002/mgg3.2059 36181358 10.1002/mgg3.2059 PMC9834195

[95] Grady JP Campbell G Ratnaike T Blakely EL Falkous G Nesbitt V Schaefer AM McNally RJ Gorman GS Taylor RW. Disease progression in patients with single, large-scale mitochondrial DNA deletions. Brain. 2014;137:323–334. doi: 10.1093/brain/awt321 24277717 10.1093/brain/awt321 PMC3914470

[96] Pan X Wang Y Liu N Luo X Sutton VR Craigen WJ Sun Q. Detecting mitochondrial electron transport chain enzyme defects in low-heteroplasmy single large-scale mtDNA deletion syndromes (SLSMDSs). Mol Genet Metab. 2025;146:109260. doi: 10.1016/j.ymgme.2025.109260 41086592 10.1016/j.ymgme.2025.109260

[97] Lechuga-Vieco AV Latorre-Pellicer A Calvo E Torroja C Pellico J Acín-Pérez R García-Gil ML Santos A Bagwan N Bonzon-Kulichenko E. Heteroplasmy of wild-type mitochondrial DNA variants in mice causes metabolic heart disease with pulmonary hypertension and frailty. Circulation. 2022;145:1084–1101. doi: 10.1161/CIRCULATIONAHA.121.056286 35236094 10.1161/CIRCULATIONAHA.121.056286 PMC8969846

[98] Schönmehl R Mendelsohn DH Winter L Pabel S Niedermair T Evert K Cheung WH Wong RMY Schmitt VH Keller K. Comparative analysis of mitochondria surrounding the intercalated discs in heart diseases-an ultrastructural pilot study. Int J Mol Sci. 2024;25:7644. doi: 10.3390/ijms25147644 39062885 10.3390/ijms25147644 PMC11277158

[99] Doenst T Nguyen TD Abel ED. Cardiac metabolism in heart failure: implications beyond ATP production. Circ Res. 2013;113:709–724. doi: 10.1161/CIRCRESAHA.113.300376 23989714 10.1161/CIRCRESAHA.113.300376 PMC3896379

[100] Yonova-Doing E Calabrese C Gomez-Duran A Schon K Wei W Karthikeyan S Chinnery PF Howson JMM. An atlas of mitochondrial DNA genotype-phenotype associations in the UK Biobank. Nat Genet. 2021;53:982–993. doi: 10.1038/s41588-021-00868-1 34002094 10.1038/s41588-021-00868-1 PMC7611844

[101] Koller A Filosi M Weissensteiner H Fazzini F Gorski M Pattaro C Schönherr S Forer L Herold JM Stark KJ. Nuclear and mitochondrial genetic variants associated with mitochondrial DNA copy number. Sci Rep. 2024;14:2083. doi: 10.1038/s41598-024-52373-0 38267512 10.1038/s41598-024-52373-0 PMC10808213

[102] Ashar FN Zhang Y Longchamps RJ Lane J Moes A Grove ML Mychaleckyj JC Taylor KD Coresh J Rotter JI. Association of mitochondrial DNA copy number with cardiovascular disease. JAMA Cardiol. 2017;2:1247–1255. doi: 10.1001/jamacardio.2017.3683 29049454 10.1001/jamacardio.2017.3683 PMC5710361

[103] Hong YS Longchamps RJ Zhao D Castellani CA Loehr LR Chang PP Matsushita K Grove ML Boerwinkle E Arking DE. Mitochondrial DNA copy number and incident heart failure: the Atherosclerosis Risk in Communities (ARIC) study. Circulation. 2020;141:1823–1825. doi: 10.1161/CIRCULATIONAHA.120.046001 32479199 10.1161/CIRCULATIONAHA.120.046001 PMC7295435

[104] Zhao D Bartz TM Sotoodehnia N Post WS Heckbert SR Alonso A Longchamps RJ Castellani CA Hong YS Rotter JI. Mitochondrial DNA copy number and incident atrial fibrillation. BMC Med. 2020;18:246. doi: 10.1186/s12916-020-01715-6 32933497 10.1186/s12916-020-01715-6 PMC7493408

[105] Wei R Ni Y Bazeley P Grandhi S Wang J Li ST Hazen SL Wilson Tang WH LaFramboise T. Mitochondrial DNA content is linked to cardiovascular disease patient phenotypes. J Am Heart Assoc. 2021;10:e018776. doi: 10.1161/JAHA.120.018776 33533264 10.1161/JAHA.120.018776 PMC7955324

[106] Li X Liu X Chen X Wang Y Wu S Li F Su Y Chen L Xiao J Ma J. Leukocyte mitochondrial DNA copy number and cardiovascular disease: a systematic review and meta-analysis of cohort studies. iScience. 2024;27:110522. doi: 10.1016/j.isci.2024.110522 39220264 10.1016/j.isci.2024.110522 PMC11363494

[107] Stoevesandt D Schlitt A Röntgen P Kraya T. Cardiac manifestations in adult MELAS syndrome (mitochondrial encephalomyopathy with lactic acidosis and stroke-like episodes syndrome)—a cross-sectional study. Orphanet J Rare Dis. 2025;20:62. doi: 10.1186/s13023-025-03534-5 39930534 10.1186/s13023-025-03534-5 PMC11812182

[108] Brambilla A Favilli S Olivotto I Calabri GB Porcedda G De Simone L Procopio E Pasquini E Donati MA. Clinical profile and outcome of cardiac involvement in MELAS syndrome. Int J Cardiol. 2019;276:14–19. doi: 10.1016/j.ijcard.2018.10.051 30482630 10.1016/j.ijcard.2018.10.051

[109] Bietenbeck M Florian AR Meier C Holtstiege V Chatzantonis G Yilmaz A. P5274 characterization of cardiac involvement in patients with MELAS syndrome in comparison to HCM patients by conventional LGE imaging and novel T1-mapping. Eur Heart J. 2019;40(Suppl_1):ehz746.0245. doi: 10.1093/eurheartj/ehz746.0245

[110] Hsu YHR Yogasundaram H Parajuli N Valtuille L Sergi C Oudit GY. MELAS syndrome and cardiomyopathy: linking mitochondrial function to heart failure pathogenesis. Heart Fail Rev. 2015;21:103–116. doi: 10.1007/s10741-015-9524-5 10.1007/s10741-015-9524-5 26712328

[111] Liu JY Numan MT Koenig MK. Mitochondrial encephalomyopathy, lactic acidosis, and stroke-like episodes (MELAS) and m.3243A>G mutations have higher prevalence of Wolff-Parkinson-White syndrome [abstract 12838]. Circulation. 2023;148(Suppl_1):A12838. doi: 10.1161/circ.148.suppl_1.12838

[112] Catteruccia M Sauchelli D Della Marca G Primiano G Cuccagna C Bernardo D Leo M Camporeale A Sanna T Cianfoni A. “Myo-cardiomyopathy” is commonly associated with the A8344G “MERRF” mutation. J Neurol. 2015;262:701–710. doi: 10.1007/s00415-014-7632-0 25559684 10.1007/s00415-014-7632-0

[113] Vallance HD Jeven G Wallace DC Brown MD. A case of sporadic infantile histiocytoid cardiomyopathy caused by the A8344G (MERRF) mitochondrial DNA mutation. Pediatr Cardiol. 2004;25:538–540. doi: 10.1007/s00246-003-0446-y 15164143 10.1007/s00246-003-0446-y

[114] Sofou K de Coo IFM Ostergaard E Isohanni P Naess K De Meirleir L Tzoulis C Uusimaa J Lönnqvist T Bindoff LA. Phenotype-genotype correlations in Leigh syndrome: new insights from a multicentre study of 96 patients. J Med Genet. 2018;55:21–27. doi: 10.1136/jmedgenet-2017-104891 29101127 10.1136/jmedgenet-2017-104891

[115] Imai-Okazaki A Kishita Y Kohda M Mizuno Y Fushimi T Matsunaga A Yatsuka Y Hirata T Harashima H Takeda A. Cardiomyopathy in children with mitochondrial disease: prognosis and genetic background. Int J Cardiol. 2019;279:115–121. doi: 10.1016/j.ijcard.2019.01.017 30642647 10.1016/j.ijcard.2019.01.017

[116] Liu Y Huang Y Xu C An P Luo Y Jiao L Luo J Li Y. Mitochondrial dysfunction and therapeutic perspectives in cardiovascular diseases. Int J Mol Sci. 2022;23:16053. doi: 10.3390/ijms232416053 36555691 10.3390/ijms232416053 PMC9788331

[117] Chen YR Zweier JL. Cardiac mitochondria and reactive oxygen species generation. Circ Res. 2014;114:524–537. doi: 10.1161/CIRCRESAHA.114.300559 24481843 10.1161/CIRCRESAHA.114.300559 PMC4118662

[118] Ide T Tsutsui H Kinugawa S Utsumi H Kang D Hattori N Uchida K Arimura K Egashira K Takeshita A. Mitochondrial electron transport complex I is a potential source of oxygen free radicals in the failing myocardium. Circ Res. 1999;85:357–363. doi: 10.1161/01.res.85.4.357 10455064 10.1161/01.res.85.4.357

[119] Ide T Tsutsui H Kinugawa S Suematsu N Hayashidani S Ichikawa K Utsumi H Machida Y Egashira K Takeshita A. Direct evidence for increased hydroxyl radicals originating from superoxide in the failing myocardium. Circ Res. 2000;86:152–157. doi: 10.1161/01.res.86.2.152 10666410 10.1161/01.res.86.2.152

[120] Ide T Tsutsui H Hayashidani S Kang D Suematsu N Nakamura K Utsumi H Hamasaki N Takeshita A. Mitochondrial DNA damage and dysfunction associated with oxidative stress in failing hearts after myocardial infarction. Circ Res. 2001;88:529–535. doi: 10.1161/01.res.88.5.529 11249877 10.1161/01.res.88.5.529

[121] Weissman D Maack C. Redox signaling in heart failure and therapeutic implications. Free Radic Biol Med. 2021;171:345–364. doi: 10.1016/j.freeradbiomed.2021.05.013 34019933 10.1016/j.freeradbiomed.2021.05.013

[122] Erickson JR Joiner ML Guan X Kutschke W Yang J Oddis CV Bartlett RK Lowe JS O’Donnell SE Aykin-Burns N. A dynamic pathway for calcium-independent activation of CaMKII by methionine oxidation. Cell. 2008;133:462–474. doi: 10.1016/j.cell.2008.02.048 18455987 10.1016/j.cell.2008.02.048 PMC2435269

[123] Wagner S Dybkova N Rasenack ECL Jacobshagen C Fabritz L Kirchhof P Maier SKG Zhang T Hasenfuss G Brown JH. Ca^2+^/calmodulin-dependent protein kinase II regulates cardiac Na^+^ channels. J Clin Invest. 2006;116:3127–3138. doi: 10.1172/JCI26620 17124532 10.1172/JCI26620 PMC1654201

[124] Aksentijevic D Sedej S Fauconnier J Paillard M Abdellatif M Streckfuss-Bömeke K Ventura-Clapier R van der Velden J de Boer RA Bertero E. Mechano-energetic uncoupling in heart failure. Nat Rev Cardiol. 2025;22:773–797. doi: 10.1038/s41569-025-01167-6 40544170 10.1038/s41569-025-01167-6

[125] Gao F Liang T Lu YW Fu X Dong X Pu L Hong T Zhou Y Zhang Y Liu N. A defect in mitochondrial protein translation influences mitonuclear communication in the heart. Nat Commun. 2023;14:1595. doi: 10.1038/s41467-023-37291-5 36949106 10.1038/s41467-023-37291-5 PMC10033703

[126] Bakovic P Mirosevic V Svagusa T Sepac A Kulic A Milicic D Gasparovic H Rudez I Urlic M Sikiric S. Reduced expression of UPRmt proteins HSP10, HSP60, HTRA2, OMA1, SPG7, and YME1L is associated with accelerated heart failure in humans. Biomedicines. 2025;13:1142. doi: 10.3390/biomedicines13051142 40426970 10.3390/biomedicines13051142 PMC12108656

[127] Xiang M Yang M Zhang L Ouyang X Sarapultsev A Luo S Hu D. Mitochondrial DNA dysfunction in cardiovascular diseases: a novel therapeutic target. Antioxidants (Basel). 2025;14:1138. doi: 10.3390/antiox14091138 41009042 10.3390/antiox14091138 PMC12466557

[128] Zong Y Li H Liao P Chen L Pan Y Zheng Y Zhang C Liu D Zheng M Gao J. Mitochondrial dysfunction: mechanisms and advances in therapy. Signal Transduct Target Ther. 2024;9:124. doi: 10.1038/s41392-024-01839-8 38744846 10.1038/s41392-024-01839-8 PMC11094169

[129] Zhou H Wang X Xu T Gan D Ma Z Zhang H Zhang J Zeng Q Xu D. PINK1-mediated mitophagy attenuates pathological cardiac hypertrophy by suppressing the mtDNA release-activated cGAS-STING pathway. Cardiovasc Res. 2025;121:128–142. doi: 10.1093/cvr/cvae238 39498806 10.1093/cvr/cvae238

[130] Papageorgiou A Sofiou FI Lembessis P Traikov LL Karela NR Angouras DC Philippou A. Mitochondrial mutations in cardiovascular diseases: preliminary findings. Genes (Basel). 2024;15:1442. doi: 10.3390/genes15111442 39596642 10.3390/genes15111442 PMC11593694

[131] Calabrese C Pyle A Griffin H Coxhead J Hussain R Braund PS Li L Burgess A Munroe PB Little L. Heteroplasmic mitochondrial DNA variants in cardiovascular diseases. PLoS Genet. 2022;18:e1010068. doi: 10.1371/journal.pgen.1010068 35363781 10.1371/journal.pgen.1010068 PMC9007378

[132] Mancuso M Orsucci D Angelini C Bertini E Carelli V Comi GP Minetti C Moggio M Mongini T Servidei S. Phenotypic heterogeneity of the 8344A>G mtDNA “MERRF” mutation. Neurology. 2013;80:2049–2054. doi: 10.1212/WNL.0b013e318294b44c 23635963 10.1212/WNL.0b013e318294b44c

[133] Yokomizo T Suda T. Development of the hematopoietic system: expanding the concept of hematopoietic stem cell-independent hematopoiesis. Trends Cell Biol. 2024;34:161–172. doi: 10.1016/j.tcb.2023.06.007 37481335 10.1016/j.tcb.2023.06.007

[134] Rajasimha HK Chinnery PF Samuels DC. Selection against pathogenic mtDNA mutations in a stem cell population leads to the loss of the 3243A-->G mutation in blood. Am J Hum Genet. 2008;82:333–343. doi: 10.1016/j.ajhg.2007.10.007 18252214 10.1016/j.ajhg.2007.10.007 PMC2427290

[135] Frederiksen AL Andersen PH Kyvik KO Jeppesen TD Vissing J Schwartz M. Tissue specific distribution of the 3243A->G mtDNA mutation. J Med Genet. 2006;43:671–677. doi: 10.1136/jmg.2005.039339 16490799 10.1136/jmg.2005.039339 PMC2564591

[136] Grady JP Pickett SJ Ng YS Alston CL Blakely EL Hardy SA Feeney CL Bright AA Schaefer AM Gorman GS. mtDNA heteroplasmy level and copy number indicate disease burden in m.3243A>G mitochondrial disease. EMBO Mol Med. 2018;10:e8262. doi: 10.15252/emmm.201708262 29735722 10.15252/emmm.201708262 PMC5991564

[137] Rahman S Poulton J Marchington D Suomalainen A. Decrease of 3243 A→G mtDNA mutation from blood in MELAS syndrome: a longitudinal study. Am J Hum Genet. 2001;68:238–240. doi: 10.1086/316930 11085913 10.1086/316930 PMC1234919

[138] Scott Binder M Roda RH Corse AM Sidhu S Stewart S Barth AS. Prevalence of heart disease in patients with mitochondrial abnormalities on skeletal muscle biopsy. Ann Clin Transl Neurol. 2021;8:825–830. doi: 10.1002/acn3.51327 33638621 10.1002/acn3.51327 PMC8045917

[139] du Fay de Lavallaz J Prepoudis A Wendebourg MJ Kesenheimer E Kyburz D Daikeler T Haaf P Wanschitz J Löscher WN Schreiner B; BASEL XII Investigators. Skeletal muscle disorders: a noncardiac source of cardiac troponin T. Circulation. 2022;145:1764–1779. doi: 10.1161/CIRCULATIONAHA.121.058489 35389756 10.1161/CIRCULATIONAHA.121.058489 PMC10069758

[140] Jaffe AS Vasile VC Milone M Saenger AK Olson KN Apple FS. Diseased skeletal muscle: a noncardiac source of increased circulating concentrations of cardiac troponin T. J Am Coll Cardiol. 2011;58:1819–1824. doi: 10.1016/j.jacc.2011.08.026 21962825 10.1016/j.jacc.2011.08.026

[141] Dunnigan A Staley NA Smith SA Ella Pierpont M Judd D Benditt DG Woodrow Benson D. Cardiac and skeletal muscle abnormalities in cardiomyopathy: comparison of patients with ventricular tachycardia or congestive heart failure. J Am Coll Cardiol. 1987;10:608–618. doi: 10.1016/s0735-1097(87)80204-8 3624667 10.1016/s0735-1097(87)80204-8

[142] Calloway CD Reynolds RL Herrin GL Anderson WW. The frequency of heteroplasmy in the HVII region of mtDNA differs across tissue types and increases with age. Am J Hum Genet. 2000;66:1384–1397. doi: 10.1086/302844 10739761 10.1086/302844 PMC1288202

[143] Naue J Hörer S Sänger T Strobl C Hatzer-Grubwieser P Parson W Lutz-Bonengel S. Evidence for frequent and tissue-specific sequence heteroplasmy in human mitochondrial DNA. Mitochondrion. 2015;20:82–94. doi: 10.1016/j.mito.2014.12.002 25526677 10.1016/j.mito.2014.12.002

[144] Cooper LT Baughman KL Feldman AM Frustaci A Jessup M Kuhl U Levine GN Narula J Starling RC Towbin J. The role of endomyocardial biopsy in the management of cardiovascular disease: a scientific statement from the American Heart Association, the American College of Cardiology, and the European Society of Cardiology Endorsed by the Heart Failure Society of America and the Heart Failure Association of the European Society of Cardiology. Eur Heart J. 2007;28:3076–3093. doi: 10.1093/eurheartj/ehm456 17959624 10.1093/eurheartj/ehm456

[145] Maruo Y Ueda Y Murayama K Takeda A. A case report of Leigh syndrome diagnosed by endomyocardial biopsy. Eur Heart J Case Rep. 2021;5:ytaa582. doi: 10.1093/ehjcr/ytaa582 33644659 10.1093/ehjcr/ytaa582 PMC7898571

[146] Katzmann JL Schlattmann P Rigopoulos AG Noutsias E Bigalke B Pauschinger M Tschope C Sedding D Schulze PC Noutsias M. Meta-analysis on the immunohistological detection of inflammatory cardiomyopathy in endomyocardial biopsies. Heart Fail Rev. 2020;25:277–294. doi: 10.1007/s10741-019-09835-9 31396762 10.1007/s10741-019-09835-9

[147] Peretto G Sala S Rizzo S De Luca G Campochiaro C Sartorelli S Benedetti G Palmisano A Esposito A Tresoldi M. Arrhythmias in myocarditis: state of the art. Heart Rhythm. 2019;16:793–801. doi: 10.1016/j.hrthm.2018.11.024 30476544 10.1016/j.hrthm.2018.11.024

[148] Bennett MK Gilotra NA Harrington C Rao S Dunn JM Freitag TB Halushka MK Russell SD. Evaluation of the role of endomyocardial biopsy in 851 patients with unexplained heart failure from 2000-2009. Circ Heart Fail. 2013;6:676–684. doi: 10.1161/CIRCHEARTFAILURE.112.000087 23733916 10.1161/CIRCHEARTFAILURE.112.000087

[149] Shields RC Tazelaar HD Berry GJ Cooper LT. The role of right ventricular endomyocardial biopsy for idiopathic giant cell myocarditis. J Card Fail. 2002;8:74–78. doi: 10.1054/jcaf.2002.32196 12016630 10.1054/jcaf.2002.32196

[150] González MDM Ramos A Aluja MP Santos C. Sensitivity of mitochondrial DNA heteroplasmy detection using next generation sequencing. Mitochondrion. 2020;50:88–93. doi: 10.1016/j.mito.2019.10.006 31669622 10.1016/j.mito.2019.10.006

[151] Dierckxsens N Mardulyn P Smits G. Unraveling heteroplasmy patterns with NOVOPlasty. NAR Genom Bioinform. 2020;2:lqz011. doi: 10.1093/nargab/lqz011 33575563 10.1093/nargab/lqz011 PMC7671380

[152] Just RS Irwin JA Parson W. Mitochondrial DNA heteroplasmy in the emerging field of massively parallel sequencing. Forensic Sci Int Genet. 2015;18:131–139. doi: 10.1016/j.fsigen.2015.05.003 26009256 10.1016/j.fsigen.2015.05.003 PMC4550493

[153] Li M Schönberg A Schaefer M Schroeder R Nasidze I Stoneking M. Detecting heteroplasmy from high-throughput sequencing of complete human mitochondrial DNA genomes. Am J Hum Genet. 2010;87:237–249. doi: 10.1016/j.ajhg.2010.07.014 20696290 10.1016/j.ajhg.2010.07.014 PMC2917713

[154] Weissensteiner H Forer L Fuchsberger C Schöpf B Kloss-Brandstätter A Specht G Kronenberg F Schönherr S. mtDNA-Server: next-generation sequencing data analysis of human mitochondrial DNA in the cloud. Nucleic Acids Res. 2016;44:W64–W69. doi: 10.1093/nar/gkw247 27084948 10.1093/nar/gkw247 PMC4987870

[155] Marquis J Lefebvre G Kourmpetis YAI Kassam M Ronga F De Marchi U Wiederkehr A Descombes P. MitoRS, a method for high throughput, sensitive, and accurate detection of mitochondrial DNA heteroplasmy. BMC Genomics. 2017;18:326. doi: 10.1186/s12864-017-3695-5 28441938 10.1186/s12864-017-3695-5 PMC5405551

[156] Frascarelli C Zanetti N Nasca A Izzo R Lamperti C Lamantea E Legati A Ghezzi D. Nanopore long-read next-generation sequencing for detection of mitochondrial DNA large-scale deletions. Front Genet. 2023;14:1089956. doi: 10.3389/fgene.2023.1089956 37456669 10.3389/fgene.2023.1089956 PMC10344361

[157] Liu YJ Sulc J Auwerx J. Mitochondrial genetics, signalling and stress responses. Nat Cell Biol. 2025;27:393–407. doi: 10.1038/s41556-025-01625-w 40065146 10.1038/s41556-025-01625-w

[158] Hsieh YH Kautz P Nitsch L Giguelay AM Liebold J Dimitrova V Contreras Castillo S Jungen F Zsurka G Trombly G. Single-cell multi-omic analysis of mitochondrial mutational mosaicism and dynamics. Nat Commun. 2026;17:2532. doi: 10.1038/s41467-026-70399-y 41839886 10.1038/s41467-026-70399-y PMC12996611

[159] O’Hara R Tedone E Ludlow A Huang E Arosio B Mari D Shay JW. Quantitative mitochondrial DNA copy number determination using droplet digital PCR with single-cell resolution. Genome Res. 2019;29:1878–1888. doi: 10.1101/gr.250480.119 31548359 10.1101/gr.250480.119 PMC6836731

[160] Galera‐Monge T Zurita‐Díaz F Garesse R Gallardo ME. The mutation m.13513G>A impairs cardiac function, favoring a neuroectoderm commitment, in a mutant‐load dependent way. J Cell Physiol. 2019;234:19511–19522. doi: 10.1002/jcp.28549 30950033 10.1002/jcp.28549

[161] Ma H Folmes CDL Wu J Morey R Mora-Castilla S Ocampo A Ma L Poulton J Wang X Ahmed R. Metabolic rescue in pluripotent cells from patients with mtDNA disease. Nature. 2015;524:234–238. doi: 10.1038/nature14546 26176921 10.1038/nature14546

[162] Chou SJ Tseng WL Chen CT Lai YF Chien CS Chang YL Lee HC Wei YH Chiou SH. Impaired ROS scavenging system in human induced pluripotent stem cells generated from patients with MERRF syndrome. Sci Rep. 2016;6:23661. doi: 10.1038/srep23661 27025901 10.1038/srep23661 PMC4812254

[163] Folmes CDL Martinez-Fernandez A Perales-Clemente E Li X Mcdonald A Oglesbee D Hrstka SC Perez-Terzic C Terzic A Nelson TJ. Disease-causing mitochondrial heteroplasmy segregated within induced pluripotent stem cell clones derived from a patient with MELAS. Stem Cells. 2013;31:1298–1308. doi: 10.1002/stem.1389 23553816 10.1002/stem.1389 PMC3706526

[164] Klein Gunnewiek TM Van Hugte EJH Frega M Guardia GS Foreman K Panneman D Mossink B Linda K Keller JM Schubert D. m.3243A>G-induced mitochondrial dysfunction impairs human neuronal development and reduces neuronal network activity and synchronicity. Cell Rep. 2020;31:107538. doi: 10.1016/j.celrep.2020.107538 32320658 10.1016/j.celrep.2020.107538

[165] Yokota M Hatakeyama H Ono Y Kanazawa M Goto Y. Mitochondrial respiratory dysfunction disturbs neuronal and cardiac lineage commitment of human iPSCs. Cell Death Dis. 2017;8:e2551–e2551. doi: 10.1038/cddis.2016.484 28079893 10.1038/cddis.2016.484 PMC5386384

[166] Mavraki E Labrum R Sergeant K Alston CL Woodward C Smith C Knowles CVY Patel Y Hodsdon P Baines JP. Genetic testing for mitochondrial disease: the United Kingdom best practice guidelines. Eur J Hum Genet. 2023;31:148–163. doi: 10.1038/s41431-022-01249-w 36513735 10.1038/s41431-022-01249-w PMC9905091

[167] Yang X Pabon L Murry CE. Engineering adolescence: maturation of human pluripotent stem cell-derived cardiomyocytes. Circ Res. 2014;114:511–523. doi: 10.1161/CIRCRESAHA.114.300558 24481842 10.1161/CIRCRESAHA.114.300558 PMC3955370

[168] Malan D Gallo MP Geddo F Levi R Querio G. Metabolic maturation in hiPSC-derived cardiomyocytes: emerging strategies for inducing the adult cardiac phenotype. Pharmaceuticals (Basel). 2025;18:1133. doi: 10.3390/ph18081133 40872524 10.3390/ph18081133 PMC12389403

[169] Gao Z Zhou F Mu J. Research progress towards the effects of fatty acids on the differentiation and maturation of human induced pluripotent stem cells into cardiomyocytes. Rev Cardiovasc Med. 2023;24:69. doi: 10.31083/j.rcm2403069 39077493 10.31083/j.rcm2403069 PMC11264038

[170] Gammage PA Gaude E Van Haute L Rebelo-Guiomar P Jackson CB Rorbach J Pekalski ML Robinson AJ Charpentier M Concordet JP. Near-complete elimination of mutant mtDNA by iterative or dynamic dose-controlled treatment with mtZFNs. Nucleic Acids Res. 2016;44:7804–7816. doi: 10.1093/nar/gkw676 27466392 10.1093/nar/gkw676 PMC5027515

[171] Gammage PA Rorbach J Vincent AI Rebar EJ Minczuk M. Mitochondrially targeted ZFNs for selective degradation of pathogenic mitochondrial genomes bearing large-scale deletions or point mutations. EMBO Mol Med. 2014;6:458–466. doi: 10.1002/emmm.201303672 24567072 10.1002/emmm.201303672 PMC3992073

[172] Bacman SR Williams SL Duan D Moraes CT. Manipulation of mtDNA heteroplasmy in all striated muscles of newborn mice by AAV9-mediated delivery of a mitochondria-targeted restriction endonuclease. Gene Ther. 2012;19:1101–1106. doi: 10.1038/gt.2011.196 22130448 10.1038/gt.2011.196 PMC3410960

[173] Bacman SR Williams SL Hernandez D Moraes CT. Modulating mtDNA heteroplasmy by mitochondria-targeted restriction endonucleases in a ‘differential multiple cleavage-site’ model. Gene Ther. 2007;14:1309–1318. doi: 10.1038/sj.gt.3302981 17597792 10.1038/sj.gt.3302981 PMC2771437

[174] Bayona-Bafaluy MP Blits B Battersby BJ Shoubridge EA Moraes CT. Rapid directional shift of mitochondrial DNA heteroplasmy in animal tissues by a mitochondrially targeted restriction endonuclease. Proc Natl Acad Sci USA. 2005;102:14392–14397. doi: 10.1073/pnas.0502896102 16179392 10.1073/pnas.0502896102 PMC1242285

[175] Yang Y Wu H Kang X Liang Y Lan T Li T Tan T Peng J Zhang Q An G. Targeted elimination of mutant mitochondrial DNA in MELAS-iPSCs by mitoTALENs. Protein Cell. 2018;9:283–297. doi: 10.1007/s13238-017-0499-y 29318513 10.1007/s13238-017-0499-y PMC5829275

[176] Yahata N Matsumoto Y Omi M Yamamoto N Hata R. TALEN-mediated shift of mitochondrial DNA heteroplasmy in MELAS-iPSCs with m.13513G>A mutation. Sci Rep. 2017;7:15557. doi: 10.1038/s41598-017-15871-y 29138463 10.1038/s41598-017-15871-y PMC5686150

[177] Bacman SR Williams SL Pinto M Peralta S Moraes CT. Specific elimination of mutant mitochondrial genomes in patient-derived cells by mitoTALENs. Nat Med. 2013;19:1111–1113. doi: 10.1038/nm.3261 23913125 10.1038/nm.3261 PMC4153471

[178] Bacman SR Williams SL Garcia S Moraes CT. Organ-specific shifts in mtDNA heteroplasmy following systemic delivery of a mitochondria-targeted restriction endonuclease. Gene Ther. 2010;17:713–720. doi: 10.1038/gt.2010.25 20220783 10.1038/gt.2010.25 PMC3175591

[179] Mok BY de Moraes MH Zeng J Bosch DE Kotrys AV Raguram A Hsu FS Radey MC Peterson SB Mootha VK. A bacterial cytidine deaminase toxin enables CRISPR-free mitochondrial base editing. Nature. 2020;583:631–637. doi: 10.1038/s41586-020-2477-4 32641830 10.1038/s41586-020-2477-4 PMC7381381

[180] Gammage PA Moraes CT Minczuk M. Mitochondrial genome engineering: the revolution may not be CRISPR-Ized. Trends Genet. 2018;34:101–110. doi: 10.1016/j.tig.2017.11.001 29179920 10.1016/j.tig.2017.11.001 PMC5783712

[181] Silva-Pinheiro P Nash PA Van Haute L Mutti CD Turner K Minczuk M. In vivo mitochondrial base editing via adeno-associated viral delivery to mouse post-mitotic tissue. Nat Commun. 2022;13:750. doi: 10.1038/s41467-022-28358-w 35136065 10.1038/s41467-022-28358-w PMC8825850

[182] Cho SI Lee S Mok YG Lim K Lee J Lee JM Chung E Kim JS. Targeted A-to-G base editing in human mitochondrial DNA with programmable deaminases. Cell. 2022;185:1764.e12–1776.e12. doi: 10.1016/j.cell.2022.03.039 35472302 10.1016/j.cell.2022.03.039

[183] Borcherding N Brestoff JR. The power and potential of mitochondria transfer. Nature. 2023;623:283–291. doi: 10.1038/s41586-023-06537-z 37938702 10.1038/s41586-023-06537-z PMC11590279

[184] Brestoff JR Singh KK Aquilano K Becker LB Berridge MV Boilard E Caicedo A Crewe C Enríquez JA Gao J. Recommendations for mitochondria transfer and transplantation nomenclature and characterization. Nat Metab. 2025;7:53–67. doi: 10.1038/s42255-024-01200-x 39820558 10.1038/s42255-024-01200-x

[185] McCully JD Cowan DB Pacak CA Toumpoulis IK Dayalan H Levitsky S. Injection of isolated mitochondria during early reperfusion for cardioprotection. Am J Physiol Heart Circ Physiol. 2009;296:H94–H105. doi: 10.1152/ajpheart.00567.2008 18978192 10.1152/ajpheart.00567.2008 PMC2637784

[186] Emani SM Piekarski BL Harrild D del Nido PJ McCully JD. Autologous mitochondrial transplantation for dysfunction after ischemia-reperfusion injury. J Thorac Cardiovasc Surg. 2017;154:286–289. doi: 10.1016/j.jtcvs.2017.02.018 28283239 10.1016/j.jtcvs.2017.02.018

[187] Guariento A Piekarski BL Doulamis IP Blitzer D Ferraro AM Harrild DM Zurakowski D Del Nido PJ McCully JD Emani SM. Autologous mitochondrial transplantation for cardiogenic shock in pediatric patients following ischemia-reperfusion injury. J Thorac Cardiovasc Surg. 2021;162:992–1001. doi: 10.1016/j.jtcvs.2020.10.151 33349443 10.1016/j.jtcvs.2020.10.151

[188] Bertero E Maack C O’Rourke B. Mitochondrial transplantation in humans: “magical” cure or cause for concern? J Clin Invest. 2018;128:5191–5194. doi: 10.1172/JCI124944 30371508 10.1172/JCI124944 PMC6264628

[189] Perrier Q Lisi V Fisherwellman K Lablanche S Asthana A Orlando G Maiocchi S. Therapeutic transplantation of mitochondria and extracellular vesicles: mechanistic insights into mitochondria bioenergetics, redox signaling, and organelle dynamics in preclinical models. Free Radic Biol Med. 2025;238:473–495. doi: 10.1016/j.freeradbiomed.2025.06.040 40570985 10.1016/j.freeradbiomed.2025.06.040 PMC12382009

[190] Chen T Majerníková N Marmolejo-Garza A Trombetta-Lima M Sabogal-Guáqueta AM Zhang Y Ten Kate R Zuidema M Mulder PPMFA den Dunnen W. Mitochondrial transplantation rescues neuronal cells from ferroptosis. Free Radic Biol Med. 2023;208:62–72. doi: 10.1016/j.freeradbiomed.2023.07.034 37536459 10.1016/j.freeradbiomed.2023.07.034

[191] Jin N Zhang M Zhou L Jin S Cheng H Li X Shi Y Xiang T Zhang Z Liu Z. Mitochondria transplantation alleviates cardiomyocytes apoptosis through inhibiting AMPKα-mTOR mediated excessive autophagy. FASEB J. 2024;38:e23655. doi: 10.1096/fj.202400375R 38767449 10.1096/fj.202400375R

[192] Gammage PA Viscomi C Simard ML Costa ASH Gaude E Powell CA Van Haute L McCann BJ Rebelo-Guiomar P Cerutti R. Genome editing in mitochondria corrects a pathogenic mtDNA mutation in vivo. Nat Med. 2018;24:1691–1695. doi: 10.1038/s41591-018-0165-9 30250142 10.1038/s41591-018-0165-9 PMC6225988

[193] Nash PA Turner KM Powell CA Van Haute L Silva-Pinheiro P Bubeck F Wiedtke E Marques E Ryan DG Grimm D. Clinically translatable mitochondrial gene therapy in muscle using tandem mtZFN architecture. EMBO Mol Med. 2025;17:1222–1237. doi: 10.1038/s44321-025-00231-5 40204990 10.1038/s44321-025-00231-5 PMC12163086

[194] Shirley JL de Jong YP Terhorst C Herzog RW. Immune responses to viral gene therapy vectors. Mol Ther. 2020;28:709–722. doi: 10.1016/j.ymthe.2020.01.001 31968213 10.1016/j.ymthe.2020.01.001 PMC7054714

[195] Yang TY Braun M Lembke W McBlane F Kamerud J DeWall S Tarcsa E Fang X Hofer L Kavita U. Immunogenicity assessment of AAV-based gene therapies: an IQ consortium industry white paper. Mol Ther Methods Clin Dev. 2022;26:471–494. doi: 10.1016/j.omtm.2022.07.018 36092368 10.1016/j.omtm.2022.07.018 PMC9418752

[196] Arjomandnejad M Dasgupta I Flotte TR Keeler AM. Immunogenicity of recombinant adeno-associated virus (AAV) vectors for gene transfer. BioDrugs. 2023;37:311–329. doi: 10.1007/s40259-023-00585-7 36862289 10.1007/s40259-023-00585-7 PMC9979149

[197] Lin L Xu H Bishawi M Feng F Samy K Truskey G Barbas AS Kirk AD Brennan TV. Circulating mitochondria in organ donors promote allograft rejection. Am J Transplant. 2019;19:1917–1929. doi: 10.1111/ajt.15309 30761731 10.1111/ajt.15309 PMC6591073

[198] Vagnozzi RJ Maillet M Sargent MA Khalil H Johansen AKZ Schwanekamp JA York AJ Huang V Nahrendorf M Sadayappan S. An acute immune response underlies the benefit of cardiac stem cell therapy. Nature. 2020;577:405–409. doi: 10.1038/s41586-019-1802-2 31775156 10.1038/s41586-019-1802-2 PMC6962570

[199] Tang J Du K. Mitochondrial base editing: from principle, optimization to application. Cell Biosci. 2025;15:9. doi: 10.1186/s13578-025-01351-8 39856740 10.1186/s13578-025-01351-8 PMC11762502

[200] Tranah GJ Katzman SM Lauterjung K Yaffe K Manini TM Kritchevsky S Newman AB Harris TB Cummings SR. Mitochondrial DNA m.3243A > G heteroplasmy affects multiple aging phenotypes and risk of mortality. Sci Rep. 2018;8:11887. doi: 10.1038/s41598-018-30255-6 30089816 10.1038/s41598-018-30255-6 PMC6082898

[201] Jeong JH Kim H Hwang SH Seo CO Kim Y Lee HS Kim YG Shim J Kim YH Kim SR. Genotype and arrhythmic risk in patients with apical hypertrophic cardiomyopathy. Heart. 2025;111:859–866. doi: 10.1136/heartjnl-2024-325218 40194828 10.1136/heartjnl-2024-325218

